# Augmented Reality–Guided Decision Support in Simulated Pediatric Cardiac Arrest: A Randomized Clinical Trial

**DOI:** 10.1001/jamanetworkopen.2026.14030

**Published:** 2026-05-22

**Authors:** Johan N. Siebert, Adam Cheng, Alexandre De Masi, Ana Rajic, Sharleen K. Olanka, Marco Generelli, Jennifer Davidson, Ryan Kang, Kangsoo Kim, Pierre-Louis Rebours, Marc Ibrahim, Donovan Duncan, Isabelle Jordan, Frédéric Ehrler, Delphine S. Courvoisier, Yiqun Lin, Sergio Manzano

**Affiliations:** 1 Department of Pediatric Emergency Medicine, Geneva Children’s Hospital, Geneva University Hospitals, Geneva, Switzerland; 2 Faculty of Medicine, University of Geneva, Geneva, Switzerland; 3 Department of Pediatrics, Cumming School of Medicine, University of Calgary, Calgary, Alberta, Canada; 4 KidSIM-ASPIRE Simulation Research Program, Alberta Children’s Hospital, University of Calgary, Calgary, Alberta, Canada; 5 Educational Technologies and Learning Sciences (TECFA), Faculty of Psychology and Educational Sciences, University of Geneva, Geneva, Switzerland; 6 Department of Electrical and Software Engineering, Schulich School of Engineering, University of Calgary, Calgary, Alberta, Canada; 7 Information Systems Directorate, Geneva University Hospitals, Geneva, Switzerland; 8 Pediatric Intensive Care Unit, Alberta Children’s Hospital, University of Calgary, Calgary, Alberta, Canada; 9 Quality of Care Division, Medical Directorate, Geneva University Hospitals, Geneva, Switzerland; 10 Department of Emergency Medicine, Cumming School of Medicine, University of Calgary, Calgary, Alberta, Canada

## Abstract

**Importance:**

Guideline-adherent management of pediatric in-hospital cardiac arrest (IHCA) remains challenging, and deviations from best practices are common. Augmented-reality (AR)–enabled, role-specific decision support may improve adherence to American Heart Association (AHA) Pediatric Advanced Life Support (PALS) guidance and key performance metrics.

**Objective:**

To determine whether an AR–enhanced, role-specific decision support system improves resuscitation performance and adherence to AHA PALS guidelines during simulated pediatric IHCA.

**Design, Setting, and Participants:**

This open-label, multicenter, simulation-based randomized clinical trial was conducted from April to May 2025 at 2 tertiary pediatric emergency centers (Geneva, Switzerland and Alberta, Canada). Participants included teams of pediatric nurses and physicians.

**Intervention:**

Teams managed a standardized scenario of a 12-minute IHCA due to hyperkalemia (progressing from nonshockable to shockable rhythms) using the AR-enhanced, role-specific decision support system (intervention) or AHA PALS pocket cards (control).

**Main Outcomes and Measures:**

The primary outcome was time from recognition of loss of pulse to first epinephrine. Secondary outcomes included adherence to 3- to 5-minute epinephrine dosing intervals, time to first defibrillation, adherence to 2-minute shock and rhythm-check cycles, chest compression fraction, peri-shock pause, medication-dosing accuracy, and user experience and technology acceptance.

**Results:**

A total of 54 participants were randomized into 18 teams (18 team leaders [12 female [71%] and 36 nurses [33 female [87%]), with 9 teams (27 participants) in each group. Mean (SD) time to first epinephrine was shorter in the intervention group (97.2 [38.5] vs 113.8 [44.5] seconds; mean difference, −16.6 seconds; 95% CI, −51.3 to 17.0 seconds; *P* = .40), but this difference was not significant. For subsequent epinephrine, the intervention group improved consistency: mean (SD) deviation from the 4-minute target was 17.2 (32.5) vs 49.7 (40.3) seconds (mean difference, −32.4 seconds; 95% CI, −58.8 to −5.8 seconds; *P* = .03), with fewer guideline violations (2 of 19 participants [11%] vs 9 of 21 participants [43%]; risk difference, −0.32; 95% CI, −0.55 to −0.05; risk ratio, 0.25; 95% CI, 0.06 to 0.996; *P* = .03). Time to first defibrillation and adherence to 2-minute cycles were similar between groups. Estimates for chest compression fraction, peri-shock pauses, and medication-dosing accuracy did not suggest meaningful between-group difference. User experience and technology acceptance were favorable.

**Conclusions and Relevance:**

In this randomized clinical trial, AR support did not clearly improve time to first epinephrine in simulated pediatric cardiac arrest, with estimates compatible with both benefit and little or no effect. It improved adherence to epinephrine dosing intervals without impairing other performance domains.

**Trial Registration:**

ClinicalTrials.gov Identifier: NCT06376643

## Key Points

**Question:**

Does a multifaceted, augmented reality (AR)–enhanced, role-specific clinical decision support system improve adherence to American Heart Association (AHA) Pediatric Advanced Life Support (PALS) guidelines and key performance metrics during simulated pediatric in-hospital cardiopulmonary arrest compared with AHA PALS pocket cards?

**Findings:**

In this randomized clinical trial of 18 teams of pediatric nurses and physicians (54 participants) there was no statistically significant difference in time to first epinephrine between groups, while adherence to epinephrine dosing intervals improved with AR support.

**Meaning:**

While AR support did not clearly improve time to first epinephrine in this study, findings suggest it may improve adherence to resuscitation guidelines without impairing other performance metrics.

## Teaser

This randomized clinical trial evaluates the effect of an augmented-reality decision support tool on time to epinephrine and adherence to pediatric advanced life support guidelines during simulated in-hospital pediatric cardiac arrest.

## Introduction

Each year, thousands of patients globally experience cardiac arrest (CA) requiring cardiopulmonary resuscitation (CPR).^1^ While less common than in adults, pediatric CA is substantial, with an estimated 20 000 in-hospital CA (IHCA) and 7000 out-of-hospital CA cases annually in the US.^2^ Despite advances in training and systems of care, survival to hospital discharge remains less than 46% for IHCA and less than 16% for out-of-hospital CA.^3^ Strict adherence to American Heart Association (AHA) pediatric advanced life support (PALS) guidelines is critical for survival and neurological outcomes.^4,5^ Unfortunately, health care practitioners often struggle to consistently deliver guideline-compliant basic^6,7^ and advanced life support,^4,8,9^ with adherence of only 20% to 40% in simulated and real events.^10,11,12^ Common deviations include delayed epinephrine,^5,13^ delayed defibrillation,^9,14,15^ medication dosing errors,^4,9,16,17^ and missed reversible causes.^4,9^ These gaps reflect the high cognitive and operational demands of resuscitation.^18,19^ Team leaders coordinate complex, time-sensitive tasks.^20,21,22^ Nurses are responsible for drug preparation and administration while maintaining situational awareness.^23,24^ Communication failures, mental overload, and poor visibility of shared goals degrade care during IHCA.^25,26,27,28^

Cognitive aids aim to mitigate these challenges^29,30^ and improve adherence to guidelines and clinical performance.^31,32,33,34^ However, traditional tools such as pocket cards and single-user mobile apps that do not support team-level coordination require diverting attention from the patient care to retrieve relevant information, thus increasing mental workload and impairing communication.^35,36^ Augmented reality (AR) overlays contextual 3-dimensional holographic elements onto the user’s real-world environment, enabling simultaneous observation and interaction with both realms in an integrated fashion.^37,38^ Recent AR innovations can deliver real-time, hands-free, role-specific, individualized clinical guidance during resuscitation. However, existing evidence remains limited, heterogeneous, and largely simulation-based, with little evaluation of team-level performance and clinical outcomes.^34,37,39,40,41^ AR-based decision support has improved PALS adherence and reduced medication errors in simulation^16,17,34,37^ but has typically supported only a single practitioner, limiting shared situational awareness and overall effectiveness. It is unknown if a multiuser, role-specific decision support system improves adherence to PALS guidelines.

To address this gap, we developed an interconnected and focused mobile applications on patient care environment (InterFACE)–AR,^42^ a multifaceted stepwise clinical decision support system that leverages AR to deliver algorithm-driven, role-specific guidance to the team leader and medication nurse, while promoting team shared situational awareness via a large display. We designed this study to evaluate the effect of InterFACE-AR on time to epinephrine and adherence to AHA PALS guidelines during simulated pediatric IHCA and assessed usability and acceptance among participants.

## Methods

### Study Design and Setting

We conducted a prospective, multicenter, randomized clinical trial in a high-fidelity simulation setting designed to approximate clinical conditions between April 2 and May 8, 2025, at 2 tertiary-level pediatric hospitals (Geneva, Switzerland, and Alberta, Canada) with dedicated simulation centers and qualified pediatric clinicians. The trial followed the Consolidated Standards of Reporting Trials (CONSORT) reporting guideline^43^ with simulation-based extensions.^44,45^ The trial protocol and statistical plan are available in Supplement 1. No substantive modifications to the protocol affecting study design, outcomes, or analyses were made after trial initiation. The study was approved by SwissEthics and the Conjoint Health Research Ethics Board of the University of Calgary. All the participants provided written consent forms.

### Participants

Eligible team leaders were attending physicians, fellows, senior residents, or physician assistants from emergency medicine, pediatric emergency medicine, general pediatrics, pediatric critical care, or pediatric anesthesia. Eligible medication and charting nurses were from pediatric emergency medicine, general pediatrics, or pediatric intensive care units. Nurses were not included as team leaders. Basic life support certification was required for eligibility. Additional certifications (eg, PALS, advanced cardiovascular life support, or pediatric emergency assessment recognition and stabilization) were recorded but not required. The study excluded individuals who had prior involvement in system design, development, or usability testing. Participants were recruited based on clinical availability and scheduled into teams to reflect typical interprofessional resuscitation teams. Detailed recruitment and team assembly procedures are provided in eAppendix 1 in Supplement 2.

### Randomization

Teams were randomized 1:1 at the team level, stratified by site, using an online generator,^46^ with a block size of 2 to maintain balance. Allocation was concealed with opaque envelopes. Participants were randomized to simulated resuscitation using InterFACE-AR (intervention) (eAppendix 2 in Supplement 2) or the AHA PALS pocket reference card (control).

### Procedures and Interventions

All participants received standardized orientation to the simulation environment and roles. Both groups completed a scripted walk-through scenario before the simulation. Additional familiarization in the intervention group was limited to operational use of the system. Detailed scenario procedures, actor roles, and simulation environment are provided in eAppendices 3 and 4 in Supplement 2. The InterFACE-AR system comprised a tablet app for algorithm display and charting, a shared display presenting clinical status (including rhythm phase; elapsed time; completed, current, and upcoming tasks; and timestamps of key interventions), and AR headsets providing role-specific guidance to the team leader and medication nurse (eAppendix 2 in Supplement 2). In the intervention group, the first epinephrine dose was prompted by the algorithm, and subsequent doses were guided by a fixed 4-minute timer within the 3- to 5-minute PALS guideline window.

### Study Outcomes

The primary outcome was the time in seconds from recognition of CA to completion of first epinephrine injection. Secondary outcomes included adherence to PALS guidelines, CPR quality metrics, and medication safety. Adherence to PALS guidelines was evaluated by measuring (1) time to initiation of CPR; (2) time to administration of the first and second hyperkalemia-specific medications; (3) time to first defibrillation; (4) time to definitive airway placement; (5) medication errors, defined as deviations greater than 10% from the correct weight-based dose, based on predefined, expert consensus–based criteria^47^; (6) chest compression fraction, which should be interpreted alongside peri-shock pauses because it may be influenced by ventilation strategy (eg, 15:2 in the absence of an advanced airway), and (7) peri-shock pause durations. Additionally, for each critical task, the time interval between CA recognition and task completion was compared with the expected theoretical time targets according to PALS recommendations.^48^ Time to advanced airway placement was included as an exploratory performance metric because current PALS guidelines do not specify a recommended timing for airway placement during IHCA, and clinician preference may influence this outcome. The intervention did not mandate intubation, and prompts were contextual and aligned with algorithm progression. All time to outcomes were extracted via video review by 2 trained and calibrated expert raters (I.J. and Y.L.) (eAppendix 5 in Supplement 2). Blinding was not possible due to the nature of the intervention. Furthermore, we assessed participants’ perception of the technology via the user experience questionnaire (UEQ) and technology acceptance model instrument; both of which have supportive validity evidence.^49,50^

### Sample Size

The trial was designed with a 2-sided α of .05 and a power of 90% to detect a clinically meaningful absolute reduction of 35 seconds in the time to first epinephrine dose^5^ (primary outcome). This difference was selected based on clinical judgment informed by observational data associating delays in epinephrine administration with worse outcomes in pediatric IHCA. Prior data reported a median (IQR) time of approximately 165 (139-173) seconds.^51^ Variance estimates were derived from these data to align with the simulation-based design. Based on Greco et al^52^ computations, and using the first and third quartiles, we estimated SD as (173 − 139)/1.35 = 25.2 seconds. Assuming similar variability between groups, a sample size of 18 teams (9 per study group) was required.

### Statistical Analysis

We used descriptive statistics to summarize the demographic characteristics of participants in both intervention and control groups. The unit of analysis was the team. Each team contributed 1 observation per outcome. Continuous outcomes were summarized as means and compared between groups using bias-corrected and accelerated bootstrapping approaches and permutation tests. To account for clustering by hospital, bootstrap resampling was performed within hospitals, with teams sampled with replacement separately within each site while preserving the original site-specific sample sizes. The nonparametric approaches were selected to account for the small sample size and potential violations of normality assumptions. Dichotomous outcomes were analyzed using relative risks and risk differences with Fisher exact tests. Haldane-Anscombe^53^ correction and the Newcombe method^54^ were applied to account for small cell counts and produce accurate confidence intervals, respectively. Dispersion in repeated measures was explored descriptively and, in a post hoc analysis not prespecified in the study protocol, compared between groups using a Fligner-Killeen test. UEQ and technology acceptance model data were reported for intervention groups only. There were no missing data. Analyses were conducted using R version 4.5.0 (R Project for Statistical Computing). All tests were 2-sided with a 5% significance level.

## Results

### Participant Flow and Baseline Characteristics

**Figure 1.**
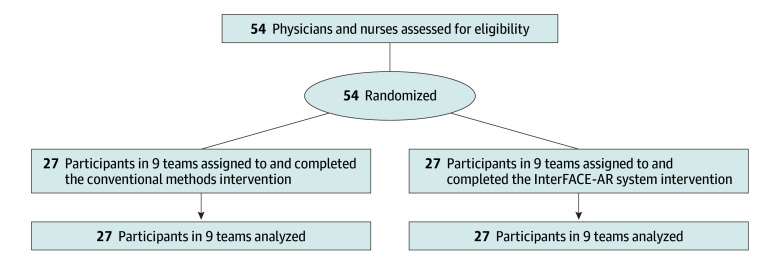
Consolidated Standards of Reporting Trials Flow Diagram InterFACE-AR indicates interconnected and focused mobile applications on patient care environment augmented reality.

A total of 54 participants were divided into 18 teams, consisting of 18 team leaders (12 female [71%]) and 36 nurses (33 female [87%]) and were included in the analyses (Figure 1). Nine teams with 27 participants were assigned to the InterFACE-AR system intervention and 9 teams with 27 participants were assigned to control, with no dropout or missing data. Assigned conditions were delivered as allocated for all participants, with full adherence to the protocol and no crossovers. Baseline characteristics were balanced in the 2 groups (Table 1), and recruitment was balanced across centers.

**Table 1.** Baseline and Clinical Characteristics of Study Participants, by Study Group

Characteristic	Study group, No. (%) (N = 54)^a^	
	Intervention group (n = 27)	Control group (n = 27)
Sex		
Male	3 (11.1)	6 (22.2)
Female	24 (88.9)	21 (77.8)
Height, mean (SD), cm	166.8 (8.4)	168.8 (7.0)
Professional role^b^		
Team leaders		
Attending physician	4 (14.8)	3 (11.1)
Resident or fellow	3 (11.1)	6 (22.2)
Physician assistant	2 (7.4)	0
Nursing staff (medication and charting roles)		
Nurse	15 (55.6)	13 (48.1)
Nurse practitioner	3 (11.1)	5 (18.5)
Instructor resuscitation certification		
Any	6 (22.2)	2 (7.4)
Time since most relevant resuscitation certification, mo^c^		
<1	4 (14.8)	1 (3.7)
1-6	5 (18.5)	7 (25.9)
7-12	0	3 (11.1)
>12	12 (44.4)	14 (51.9)
Resuscitation events involved in the past year, mean (SD), No.	1.2 (1.5)	1.8 (2.9)
Simulated resuscitation events involved in the past year, mean (SD), No.	4.1 (5.3)	2.7 (2.4)
Real CPR in the past year, mean (SD), No.	0.4 (0.6)	0.5 (0.8)
Simulated CPR in the past year, mean (SD), No.	2.5 (3.4)	1.6 (1.5)
Past experience with immersive technologies (VR or AR)		
Never	25 (92.6)	23 (85.2)
Once a month or less	2 (7.4)	3 (11.1)
Once a week to once a month	0	1 (3.7)
Experience of using AR for education		
Never	24 (88.9)	20 (74.1)
Once a month or less	2 (7.4)	7 (25.9)
Once a week to once a month	1 (3.7)	0

Abbreviations: AR, augmented reality; CPR, cardiopulmonary resuscitation; VR, virtual reality.

^a^ Percentages may not total 100 because of rounding.

^b^ Participants were assigned to predefined roles within each team. Team leaders were physicians or physician assistants. Physician assistants functioned exclusively as team leaders and were not involved in medication preparation, administration, or charting. These tasks were performed by nursing staff (nurses and nurse practitioners).

^c^ Time since most relevant resuscitation certification was defined as time since the highest level of certification held by the participant (pediatric advanced life support, advanced cardiovascular life support, or pediatric emergency assessment recognition and stabilization); for participants without advanced certification, time since basic life support certification was used. Basic life support certification was required for all participants, whereas advanced certifications were not.

### Primary Outcome

**Figure 2.**
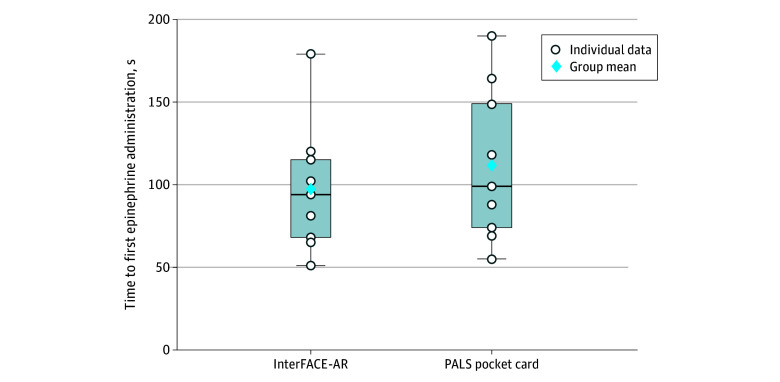
Box and Whisker Plots of Time to First Epinephrine by Study Group Box-and-whisker plots showing the distribution of time to the first epinephrine dose for teams using the interconnected and focused mobile applications on patient care environment augmented reality (InterFACE-AR) system vs a pediatric advanced life support (PALS) pocket card. The bottom edge, middle black line, and top edge of the boxes represent the 25th percentile, median, and 75th percentile values, respectively. The whiskers are plotted using the Tukey method, extending from the lower extreme (25th percentile − 1.5 × the IQR) to the upper extreme (75th percentile + 1.5 × the IQR). The blue diamond marks the mean. White dots are individual team results. Times are in seconds from scenario start.

There was no significant difference in time to first epinephrine dose between InterFACE-AR and AHA PALS pocket cards (mean [SD], 97.2 [38.5] vs 113.8 [44.5] seconds; mean difference, −16.6 seconds; 95% CI, −51.3 to 17.0 seconds; *P* = .40) (Figure 2).

### Secondary Outcomes Measures

**Figure 3.**
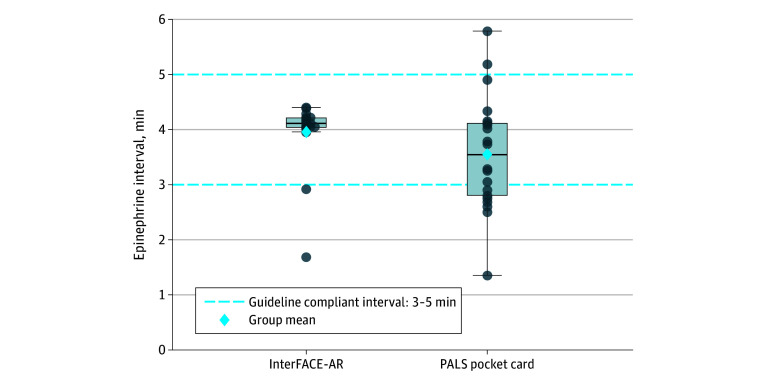
Box and Whisker Plots of Timing of Subsequent Doses of Epinephrine by Study Group Distribution of time intervals between subsequent epinephrine doses for teams using the interconnected and focused mobile applications on patient care environment augmented reality (InterFACE-AR) system (intervention) vs the American Heart Association pediatric advanced life support (PALS) pocket card (control). Each dot represents an individual interdose interval (measured from the prior dose). Box-and-whisker plots summarize the distribution, with the median shown by the horizontal line, box limits representing the IQR, and whiskers extending to the upper and lower extremes (1.5 × IQR). The blue diamond marks the mean. Dashed blue horizontal lines denote the American Heart Association guideline-recommended interval of 3 to 5 minutes. Dots outside this range represent non–guideline-compliant intervals (InterFACE-AR: 2 of 19 participants; PALS pocket card: 9 of 21 participants).

Time to completion of other critical tasks during the CA scenario, including initiation of CPR, first defibrillation attempt, advanced airway placement, and hyperkalemia-specific medications, was similar between groups (Table 2). Although time to the first occurrence of these tasks did not suggest meaningful difference between groups, adherence to prespecified PALS time targets on subsequent occurrences diverged (eFigure 1 in Supplement 2). For epinephrine, timing variability in the Interface-AR group remained tightly clustered around the target time points across successive CPR cycles, yielding a higher proportion of actions within 3- to 5-minute target windows, whereas the control group exhibited drift from AHA targets with broader dispersion (median [IQR], 213.0 [178.0-247.0] seconds vs 247 [237.5-252.5] seconds; Fligner-Killeen *P* = .002) (Figure 3 and eFigure 2 in Supplement 2).

**Table 2.** Time to Critical Tasks and CPR Quality Metrics by Study Group

Item	Time to task, mean (SD), s		Mean difference (95% CI)^a^	*P* value^b^
	Intervention group	Control group		
Teams, No.	9	9		
Time to initiate CPR	20.8 (6.6)	26.6 (12.7)	−5.8 (−14.4 to 2.3)	.26
Time to first dose of epinephrine	97.2 (38.5)	113.8 (44.5)	−16.6 (−51.3 to 17.0)	.40
Time to first defibrillation attempt after shockable rhythm onset	102.7 (28.3)	83.5 (56.0)	19.1 (−18.4 to 53.1)	.37
Time to establish advanced airway, mean (SD), s	226. 1 (191.6)	347.8 (240.0)	−121.7 (−259.2 to 27.1)	.16
Time to first hyperkalemia drug administration after diagnosis confirmation	576.7 (146.5)	593.0 (127.3)	−16.3 (−116.3 to 76.3)	.78
Time to second hyperkalemia drug administration after diagnosis confirmation	668.3 (104.4)	701.2 (56.3)	−32.9 (−97.2 to 25.9)	.54
Chest compression fraction, mean (SD), %	79.8 (16.3)	69.7 (16.6)	10.1 (−0.1 to 20.5)	.08
Peri-shock pause duration	11.6 (10.8)	17.3 (18.9)	−5.7 (−20.6 to 5.6)	.50

Abbreviation: CPR, cardiopulmonary resuscitation.

^a^ 95% CI of mean difference was determined using bias and an acceleration-corrected bootstrap.

^b^ *P* value for permutation test.

Mean (SD) deviation from the theoretical value (4 minutes) was significantly different (Interface-AR: 17.2 [32.5] seconds; PALS pocket card: 49.7 [40.3] seconds, mean difference: −32.4 seconds; 95% CI, −58.8 to −5.8 seconds; *P* = .03) (Figure 3). Guideline violations for epinephrine (ie, doses outside the recommended dosing interval of 3-5 minutes) were more frequent with the PALS cards than with AR (9 of 21 participants[43%] vs 2 of 19 participants [11%]; risk difference, −0.32; 95% CI: −0.56 to 0.05; risk ratio: 0.25; 95% CI, 0.06 to 0.996; *P* = .03) (eFigure 2 in Supplement 2).

Mean (SD) time to first shock was 102.7 (28.3) seconds with InterFACE-AR vs 83.5 (55.6) seconds with the PALS pocket card (mean difference, 19.1 seconds; 95% CI, −18.4 to 53.1 seconds; *P* = .37) (eFigure 3 in Supplement 2). The absolute deviation from the scheduled 2-minute defibrillation intervals was 21.6 seconds higher in the intervention group (47.4 seconds with InterFACE-AR vs 25.9 seconds with PALS cards; 95% CI of difference, −0.7 to 43.8 seconds; *P* = .07) (eFigures 3 and 4 in Supplement 2). Deviations from the theoretical 2-minute interval for pulse and rhythm checks were similar between groups (mean [SD] absolute deviation, 65.8 [29.7] seconds with InterFACE-AR vs 61.6 [35.8] seconds with PALS cards; mean difference, 4.3 seconds; 95% CI, −7.7 to 16.5 seconds; *P* = .49).

The analysis of chest compression fraction and peri-shock pause durations did not show any statistically significant difference between both study groups (Table 2). Medication dosing accuracy was comparable between both groups; the proportion of greater than10% dose deviations for epinephrine, amiodarone, or calcium gluconate did not differ (eTable 1 in Supplement 2).

UEQ ratings were positive across all 6 constructs (means [SDs] ranging from 1.7 [1.0] to 2.3 [0.7]), consistent with good to excellent UEQ benchmark (eTable 2 in Supplement 2). All construct means exceeded the positive threshold. Technology acceptance was high (perceived usefulness: mean [SD], 5.6 [1.4]; ease of use: mean [SD]: 5.7 [1.4]) (eTable 3 in Supplement 2). The 2 constructs were strongly correlated (r = 0.62; *P* < .001).

## Discussion

This randomized clinical trial is one of the first, to our knowledge, to evaluate an AR-enabled, multifaceted, role-specific decision support system for resuscitation teams. Our findings suggest the system did not clearly shorten time to first epinephrine dose in simulated IHCA compared with conventional care using AHA PALS pocket cards. The confidence interval around the estimated effect spanned values consistent with both a clinically meaningful reduction and little or no effect, indicating the estimate was imprecise and that modest effects in either direction cannot be excluded. Accordingly, these findings should be interpreted as preliminary, and adequately powered studies are needed to more precisely estimate the effect. There were no differences in CPR initiation, defibrillation, advanced airway placement, or treatment of reversible causes. However, the AR group maintained tighter adherence to timing targets for certain repeated tasks.

Current PALS guidelines^48^ and the International Liaison Committee on Resuscitation recommendations^55^ advise administering epinephrine as early in the resuscitation as possible for nonshockable rhythms and then every 3 to 5 minutes, but neither specifies an exact threshold for the first dose or subsequent intervals.^55,56^ In our trial, first dose epinephrine clustered around 1.5 minutes in both groups, within recommended windows^48^ and consistent with prior IHCA cohorts reporting median (IQR) times near 1 (0-4) minutes in real-world practice.^5^ Part of this timing likely reflects bedside preparation logistics in addition to recognition and decision-making. Observational pediatric studies associate each minute of delay with worse outcomes rather than identifying a single time cut point.^5,57^ The standardized simulation, highly trained participants, scripted actors, and minimized distractions and cognitive stressors likely produced near-ceiling performance, limiting detectable gains in initial actions. Under such conditions, the benefit of digital support appears greater for maintaining dosing cadence and on-time repetition of tasks than to shorten time to the first intervention by a few seconds.

This interpretation aligns with prior work showing AR devices can improve adherence to specific resuscitation steps without shortening early critical action timing.^37^ In neonatal simulation, Tsang et al^38^ similarly reported that AR-based aid improved guideline adherence and reduced critical errors but had no measurable effect on the timing of key interventions. Likewise, among emergency medicine residents, AR-assisted code-cart training did not shorten time to intubation compared with video instruction.^58^ More broadly, studies of digital cognitive aids suggest that such tools primarily enhance protocol adherence and reduce omissions during pediatric CA rather than accelerate initial actions under pressure.^34,59^ Consistent with this finding, the InterFACE-AR system was designed as a cognitive aid to support, not replace, clinician decision-making by reducing omissions and variability while preserving autonomy.

In our study, embedding the PALS algorithm in the AR workflow increased stepwise adherence and tightened the dispersion of task timing around prespecified targets. Notably, timing variability decreased over the course of the scenario in the intervention arm, yielding a higher proportion of actions within the target windows, whereas the control group exhibited progressive drift and broader dispersion from the expected time points despite ready access to the PALS pocket cards. These observations are consistent with prior studies demonstrating that structured cognitive aids and protocolized workflows reduce variability around time-critical milestones and improve on-time task execution compared with usual practices.^16,17,31^

Defibrillation timing was an exception. In the AR group, alignment with the theoretical 2-minute interval was less consistent. The workflow enforced full 2-minute CPR cycles to prioritize uninterrupted chest compressions between rhythm checks,^34^ but it did not prompt precharge. Experienced teams typically precharge 15 to 20 seconds before the target to deliver a shock at exactly 2 minutes. Here, because the AR cues shocks at the 2-minute mark, delivery drifted to around 2 minutes and 15 seconds to 2 minutes and 20 seconds. In contrast, several control teams performed an early rhythm check when they perceived a rhythm change, stopping compressions before the end of the cycle and achieving earlier defibrillation. This earlier shock in the control group is therefore, in part, a function of the InterFACE design; the system intentionally disallows rhythm checks before 2 minutes to preserve compression continuity. These findings therefore reflect differences in workflow strategies rather than imbalance in scenario conditions. Small additional delays may also have arisen if the AR timer anchored to event logging (eg, the resume CPR entry) rather than the actual resumption of chest compressions. The consistent rightward shift in the AR group suggests a systematic bias (eFigure 4 in Supplement 2). Future iterations should introduce precharge prompts and tighten timing anchors.

Finally, participants rated the AR system as usable and acceptable, with favorable UEQ scores and high technology acceptance ratings. For clinical decision support, usability and clinician acceptance are prerequisites for impact.^60^ When usability is poor, clinicians adopt workarounds,^61^ override alerts,^62^ and abandon tools,^63^ whereas user-centered design and iterative usability testing improve uptake and workflow fit.^60^ Because usability and acceptance are strong predictors of real-world adoption, these findings support integrating AR-enhanced decision support into training and, if technical and workflow constraints are addressed and safeguards are in place, into in situ care. Notably, participants rated the system positively on the UEQ dimensions of stimulation and novelty, indicating that it was perceived as engaging and motivating, which may support sustained use over time.

Overall, AR-enhanced, role-specific support improved adherence to time-critical guidance without compromising CPR quality or medication safety, supporting its use to assist resuscitation teams in high-stakes clinical environments. Implementation in clinical settings will require consideration of equipment availability, calibration, integration into clinical workflows, training, and costs. These challenges must be balanced against potential benefits in improving adherence to guidelines and reducing variability in high-risk situations.

### Limitations

This study has several limitations. First, the small sample size may have limited precision and precluded subgroup analyses. Observed variability exceeded initial assumptions, further reducing precision. Reporting individual-level data may further inform variability in performance and guide the design of future studies beyond summary measures such as means and SDs. Second, outcomes were process measures in a simulated environment and may not fully reflect real-world clinical performance. Third, although both groups received standardized orientation and practice, the intervention group required additional device familiarization. Fourth, the use of a fixed 4-minute timer for epinephrine dosing in the intervention arm may have reduced variability by design, and findings related to timing consistency should be interpreted in that context.

## Conclusions

In this randomized clinical trial, AR support did not clearly improve time to first epinephrine during simulated pediatric CA. The system improved adherence to epinephrine dosing intervals without impairing other performance measures. Clinical validation is warranted.

## Notes

Supplement 1. Trial Protocol and Statistical Analysis Plan

Supplement 2. eAppendix 1. Recruitment and team assembly eAppendix 2. The InterFACE-AR decision support system eAppendix 3. Scenario procedures, actor roles and simulation environment eAppendix 4. Cardiac arrest simulation scenario eAppendix 5. Data collection and sources eFigure 1. Timing of critical resuscitation actions relative to AHA PALS targets eFigure 2. Timing of sequential epinephrine doses and deviations from AHA recommended intervals eFigure 3. Time to first and subsequent defibrillations, by study groups eFigure 4. Timing of sequential defibrillations and deviations from AHA recommended intervals eTable 1. Medication dosing accuracy, by study group eTable 2. User experience questionnaire eTable 3. Technology acceptance model eReferences

Supplement 3. Data Sharing Statement
