# Supplementary material for: Augmented Reality–Guided Decision Support in Simulated Pediatric Cardiac Arrest: A Randomized Clinical Trial
Source: JAMA Netw Open. 2026 May 22;9(5):e2614030. doi: 10.1001/jamanetworkopen.2026.14030 (PMC13197869; doi:10.1001/jamanetworkopen.2026.14030)
Supplement: Supplement 1. — Trial Protocol and Statistical Analysis Plan [file jamanetwopen-e2614030-s001.pdf]

Augmented Reality-Guided Decision Support in Simulated Pediatric Cardiac Arrest

A Randomized Clinical Trial

Study Protocol

National Clinical Trial (NCT) Identifier Number: NCT06376643.

Registered on April 18, 2024.

Version Number: v.1.0

October 31, 2025

This reformatted version of the study protocol was created on October 31, 2025 at the request of the reviewing journal.

ROLES AND RESPONSIBILITIES

Principal Investigators:

Johan N. SIEBERT, MD[1,2]
Contact: Johan.Siebert@hug.ch

Adam Cheng, MD[3,4]
Contact: Chenger@me.com

Co-investigators:
Delphine S. COURVOISIER, PhD[2,5]; Frédéric EHRLER, PhD[6]; Yiqun LIN, MD, PhD[3]

Project Partners: Alexandre DE MASI, PhD[2] ; Kangsoo KIM, PhD[7]

Contributors:
Ana RAJIC, MS[2,8]; Sharleen K. OLANKA, MS[2,8]; Marco GENERELLI, MS[2,8]; Jennifer DAVIDSON, RN[3,4]; Minseok KANG, PhD[7]; Pierre-Louis Rebours[6]; Marc IBRAHIM[6]; Donovan DUNCAN, MD[9]; Isabelle LE GRAS JORDAN, RN[1]; Sergio MANZANO, MD[1,2]

Affiliations:
[1] Department of Pediatric Emergency Medicine, Geneva Children's Hospital, Geneva University Hospitals, 47 Avenue de la Roseraie, 1211 Geneva 14, Switzerland
[2] Faculty of Medicine, University of Geneva, 2 Rue Michel Servet, 1211 Geneva 4, Switzerland
[3] Departments of Pediatrics and Emergency Medicine, Cumming School of Medicine, University of Calgary, 2500 University Drive NW, Calgary, T2N 1N4, Alberta, Canada
[4] KidSIM-ASPIRE Simulation Research Program, Alberta Children's Hospital, University of Calgary, C4-200 28 Oki Drive NW, Calgary, T3B 6A8, Alberta, Canada
[5] Quality of Care Division, Medical directorate, Geneva University Hospitals, 4 Rue Gabrielle Perret-Gentil, 1211 Geneva 14, Switzerland
[6] Information Systems Directorate, Geneva University Hospitals, 4 Rue Gabrielle Perret-Gentil, 1211 Geneva 14, Switzerland
[7] Department of Electrical and Software Engineering, Schulich School of Engineering, University of Calgary, 2500 University Drive NW, Calgary, T2N 1N4, Alberta, Canada
[8] Educational Technologies and Learning Sciences (TECFA), Faculty of Psychology and Educational Sciences, University of Geneva, Villa Battelle (Bulding A), 7 Route de Drize, 1227 Carouge, Geneva, Switzerland
[9] Pediatric Intensive Care Unit, Alberta Children's Hospital, University of Calgary, 28 Oki Drive NW, Calgary, T3B 6A8, Alberta, Canada

Sponsor: Johan N. SIEBERT (within the meaning of the Swiss Ordinance on Clinical Trials in Human Research of 20 Sept. 2013, Art. 2)

Conflict of Interest Disclosure: Geneva University Hospitals and University of Calgary are the shared owners of the InterFACE-AR system. Drs Siebert, Cheng, Kim, Ehrler and Manzano declare individual intellectual property rights on this system. We report no other disclosures.

Funding/Support: This investigator-initiated study was funded by the Swiss National Science Foundation, grant No. 10.002.119, the Geneva University Hospitals Private Foundation, grant No RP07, and grants from the Alberta Children's Hospital Foundation, Alberta Children's Hospital Research Institute, and the Department of Pediatrics at the University of Calgary.

Role of the Funder/Sponsor: The funders had no role in the design and conduct of the study; collection, management, analysis, and interpretation of the data; preparation, review, or approval of the manuscript; and decision to submit the manuscript for publication.

Identifier Number: National Clinical Trial, NCT06376643. Registered on April 18, 2024.

Version: 1.000 Date: October 31, 2025

TABLE OF CONTENTS

SUMMARY **4**
1. INTRODUCTION **5**
1.1 Background and Rationale *5*
1.2 Previous Work Justifying This Trial *5*
1.3 Evidence Before the Current Project *6*
2. METHODS **6**
2.1 Study Objectives *6*
2.2 The InterFACE-AR Decision Support System *6*
2.3 Scientific Aims *7*
2.4 Trial Design and Setting *7*
2.5 Participants and Eligibility Criteria *8*
2.6 Recruitment Process *9*
2.7 Criteria for Discontinuing or Modifying Interventions *9*
2.8 Strategies to Improve Adherence *9*
3. TRIAL INTERVENTIONS **9**
3.1 Interventions *9*
3.2 Orientation and Simulation Scenarios *10*
4. OUTCOMES **10**
5. PARTICIPANT TIMELINE **11**
6. DATA COLLECTION **12**
6.1 Methods of Measurement *12*
6.2 Retention and Follow-up *12*
7. STATISTICAL CONSIDERATIONS **12**
7.1 Power and Sample Size Calculation *12*
7.2 Group Allocation *13*
7.3 Blinding *13*
7.4 Statistical Analysis Plan *13*
8. SAFETY ASSESSMENTS **16**
8.1 Risks to Participants *16*
8.2 Adverse Events and Serious Events *16*
8.3 Confidentiality *16*
9. ADMINISTRATIVE ASPECTS, MONITORING AND PUBLICATION **17**
9.1 Handling and Storage of Data and Documents *17*
9.2 Trial Steering Committee *17*
9.3 Monitoring and Quality Assurance *17*
9.4 Interim Analysis *17*
10. ETHICS **18**
10.1 Research Ethics Approval *18*
10.1 Protocol Amendments *18*
11. RELEVANCE AND IMPACT **18**
11.1 Clinical practice *18*
11.2 Academic learning *18*
12. REFERENCES **19**
SPIRIT 2025 CHECKLIST OF ITEMS TO ADDRESS IN A RANDOMIZED TRIAL PROTOCOL **22**

SUMMARY

Background: Each year, thousands of children experience cardiac arrests, requiring immediate and accurate resuscitation for favorable outcomes. However, adherence to American Heart Association (AHA) advanced life support guidelines remains suboptimal, hindered by cognitive and communication challenges in high-stress situations. Unfortunately, despite recurrent training, healthcare providers struggle to maintain consistent adherence to these guidelines, resulting in only 20-40% of providers achieving guideline-compliant resuscitation care. With merely 40% of children surviving to hospital discharge after experiencing in-hospital (IHCA), there is an urgent need to design, evaluate, and implement innovative strategies to enhance the provision of advanced life support care. To date, the resuscitation room environment has received little attention, with few studies exploring how modifying the clinical environment could enhance clinical performance. Supporting resuscitation teams by integrating decision support technology represents a unique opportunity. To address this problem, we will assess the InterFACE-AR system. The system aims to provide individualized, real-time, role-specific decision support to the team leader and medication nurse through optically see-through augmented reality (AR) headsets and by displaying a dynamic roadmap for patient care on a large LCD screen in the resuscitation room, controlled by a mobile tablet app.

Objective: To assess whether the use of InterFACE-AR (intervention group) improves adherence to AHA PALS guidelines, when compared to groups using the AHA PALS pocket reference card (control).

Method: An open-label, prospective, multicenter, cluster randomized clinical trial comparing InterFACE-AR to conventional methods in adherence to AHA PALS guidelines during simulated pediatric IHCA scenarios.

Participants: 54 physicians and nurses, in teams of seven, in two tertiary hospitals.

Intervention: Randomization centralized with a 1:1 ratio to either InterFACE-AR support (intervention; n=27) or conventional method (control; n=27). Each participant will undergo a 12-min standardized realistic pediatric IHCA resuscitation scenario.

Outcomes: The primary outcome is the time to first dose of epinephrine, defined as the interval in seconds from recognition of CA at the start of the scenario to completion of epinephrine injection. Secondary outcomes are times to CPR initiation, defibrillation, drug delivery, airway securing, the rate of medication errors, chest compression fraction and peri-shock pause duration. User experience (UEQ), and technology acceptance (TAM) will be also assessed among the intervention group.

Perspectives: This project has the potential to exceed current clinical practice standards and reshape resuscitation care by harnessing immersive technology and fostering interdisciplinary collaboration. Our knowledge translation strategy through publications and international organizations should ensure widespread dissemination of findings, catalyzing a transformative shift in digital health applications for pediatric resuscitation worldwide. Our project's alignment with AHA resuscitation guidelines may lead to future adaptations for adult cardiopulmonary resuscitation.

1. Introduction

1.1 Background and Rationale

Each year, thousands of children experience cardiopulmonary arrests (CA), requiring immediate and accurate resuscitation for favorable outcomes. However, adherence to American Heart Association (AHA) advanced life support guidelines remains suboptimal, hindered by cognitive and communication challenges in high-stress cardiopulmonary resuscitation (CPR) situations. To address this problem, we will assess a novel augmented reality (AR)-assisted digital health solution designed to offer personalized, real-time decision support for team leaders and medication nurses, while concurrently optimizing team situational awareness and communication by projecting a dynamic care roadmap onto a large screen in the resuscitation room, controlled via a mobile tablet app.

The solution comprises three essential digitally interconnected elements: 1) a mobile app that presents clinical algorithms alongside patient data, guiding the resuscitation team through step-by-step procedures, 2) a giant screen that displays real-time patient information and task progress for the entire resuscitation team, and 3) augmented reality headsets worn by team members, providing role-specific guidance and decision support based on data collected by the app.

In this trial, we will assess, amongst pediatric healthcare teams, whether the use of the AR-assisted tool improves adherence to AHA resuscitation guidelines and performance, when compared to groups using the AHA Paediatric Advanced Life Support (PALS) pocket reference cards (control) in an open-label, prospective, multicenter, cluster randomized (1:1 ratio) clinical trial. Resuscitation teams of physicians and nurses will be asked to manage simulated pediatric in-hospital cardiac arrest (IHCA) scenarios using either an AR-assisted tool or conventional AHA PALS pocket cards. Each team will comprise three participants assigned to the roles of 1) Team Leader (a physician), 2) Charting Nurse, and 3) Medication Nurse, with two CPR Providers, an Airway Provider, and a Bedside Provider role filled by research actors.

1.2 Previous Work Justifying This Trial

In the past, our multicenter studies have successfully influenced the use of debriefing tools for AHA instructors[1, 2], the implementation of feedback devices for CPR skills training[3-5], and the integration of CPR coaching[6-8] across AHA's life support courses (e.g. BLS, PALS, ACLS). A randomized controlled trial by our team, recognized as pioneering in a scoping review[9], showed that an AR device delivering real-time clinical guidance to the team leader during simulated pediatric IHCA reduced defibrillation dosing errors by 53% compared to AHA pocket cards[10, 11]. However, reliance on the team leader alone

increased workload and did not improve critical timings for epinephrine or defibrillation. Additionally, the system's limitations in situating the current action within the entire resuscitation process, coupled with its small size, posed major challenges to effectively displaying CPR algorithms during pediatric IHCA. Building on these lessons, we developed Guiding Pad, a mobile app used by the charting nurse to deliver interactive, role-specific guidance and cognitive aids aligned with PALS algorithms. In a single-center RCT, Guiding Pad significantly reduced time to defibrillation, decreased medication and energy dose errors, and improved adherence to PALS guidelines by ~70% compared to pocket cards[12]. Integrated with a large display - called InterFACE (Interconnected and Focused Mobile Applications in the patient **C**are **E**nvironment) - the system supports real-time team situational awareness and communication. This work aligns with the AHA's 2024 call to advance health technology[13] and supports our aim to leverage AR to improve pediatric resuscitation outcomes.

1.3 Evidence Before the Current Project

We searched PubMed, Web of Science, EMBASE and the Cochrane Library for peer-reviewed articles on AR in emergency medicine, spanning from inception to March 18, 2024, without language or age restrictions. MeSH terms "Cardiopulmonary resuscitation", "Emergency Medicine" and "Augmented reality" were explored and connected by Boolean operators. Out of 84 articles retrieved, 34 were excluded based on title and an additional 24 on abstract review. Among the 26 studies identified, three thematic categories emerged; AR in resuscitation education (n=15), prehospital and disaster medicine (n=5), and review articles (n=5). But we only identified our previous single-center trial[10] as focusing on the clinical application of AR to aid adherence to AHA PALS algorithms. Most of the studies used Google Glass and Microsoft HoloLens as AR devices, with the latter now incorporating depth sensing and human gesture recognition, representing the latest trend in AR[14, 15].

2. Methods

2.1 Study Objectives

In the proposed study, we aim to evaluate the effect of an enhanced system, InterFACE-AR (see Point 2.2 below), which significantly evolves the initial InterFACE version to leverage the capabilities of AR, on key performance metrics, including time to epinephrine and adherence to AHA guidelines during simulated pediatric IHCA scenarios. We also aimed to explore the usability and acceptance of the InterFACE-AR system amongst participants.

2.2 The InterFACE-AR Decision Support System

This system integrates three interconnected components (**Figure 1**): (1) The Guiding-Pad app, operated by the charting nurse. It displays the appropriate resuscitation algorithm and clinical status, allowing for the charting nurse to input data in real-time. (2) Clinical data is broadcast to the TeamScreen, a shared

display that provides the resuscitation team with a structured, chronological overview of the current algorithm, clinical guidance (current and next steps), and a summary of ongoing and completed tasks. (3) Simultaneously, two AR headsets (Microsoft HoloLens 2™) deliver role-specific, real-time clinical guidance to the team leader and medication nurse, such as prompts for drugs and defibrillation (for the team leader) or suggested medication and doses (for the medication nurse).

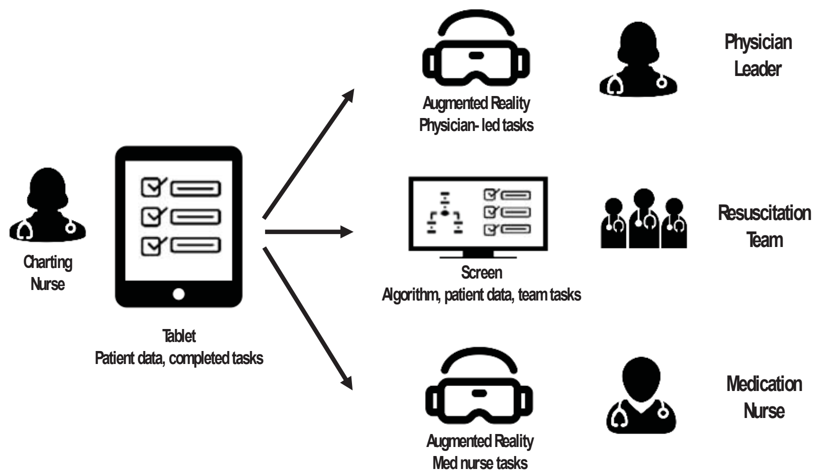

Figure 1. InterFACE-AR system components

2.3 Scientific Aims

Primary scientific aim: To determine amongst pediatric healthcare teams whether use of InterFACE-AR (intervention group) reduces the time from loss-of-pulse recognition to first epinephrine administration, compared with standard practice using the AHA PALS pocket reference cards (control).

Second scientific aim: To assess whether the use of InterFACE-AR improves resuscitation performance and adherence to AHA PALS guidelines, compared with standard practice.

2.4 Trial Design and Setting

We plan to conduct an open-label, prospective, simulation-based, multicenter, cluster randomized clinical trial at both Geneva (Switzerland) and Calgary (Canada) Children's Hospitals. Simulation-based research confers the advantage of answering research questions without risk of harm to patients[16]. The scenarios will be conducted in situ at the institutional simulation centers. We will compare team performance using the InterFACE-AR supportive tool (intervention) or conventional AHA PALS pocket reference cards (control) during standardized pediatric IHCA. Participants will be recruited to the study in teams of three, comprised of a team leader, charting nurse, and medication nurse. Additionally, four trained research actors will fill the scripted roles of CPR provider / CPR Coach (2

actors, alternating roles), an airway provider, and a Bedside Provider to make a resuscitation team of 7 providers. The actor will be trained with methodology successfully used in prior multicenter trials[17]. Participant teams will manage one CA simulation scenario (see below), using the specific trial intervention assigned during randomization. No changes will be made to the apps and devices during the study. **Figure 2** shows the trial flow chart and **Figure 3** the trial checklist. As this simulation-based study enrolls only healthcare professionals, there will be no patient or public involvement in the design, conduct, or reporting of the trial. The trial will be conducted according to appropriate guidelines[18-21] and follow the 2025 Consolidated Standards of Reporting Trials (CONSORT) reporting guideline[22]. The present trial protocol adheres to the SPIRIT 2025 checklist[23] (see **Appendix** at the end of the protocol). It is our intention to present the results at scientific congresses and to publish the results in a peer-reviewed journal, irrespective of the magnitude or direction of effect.

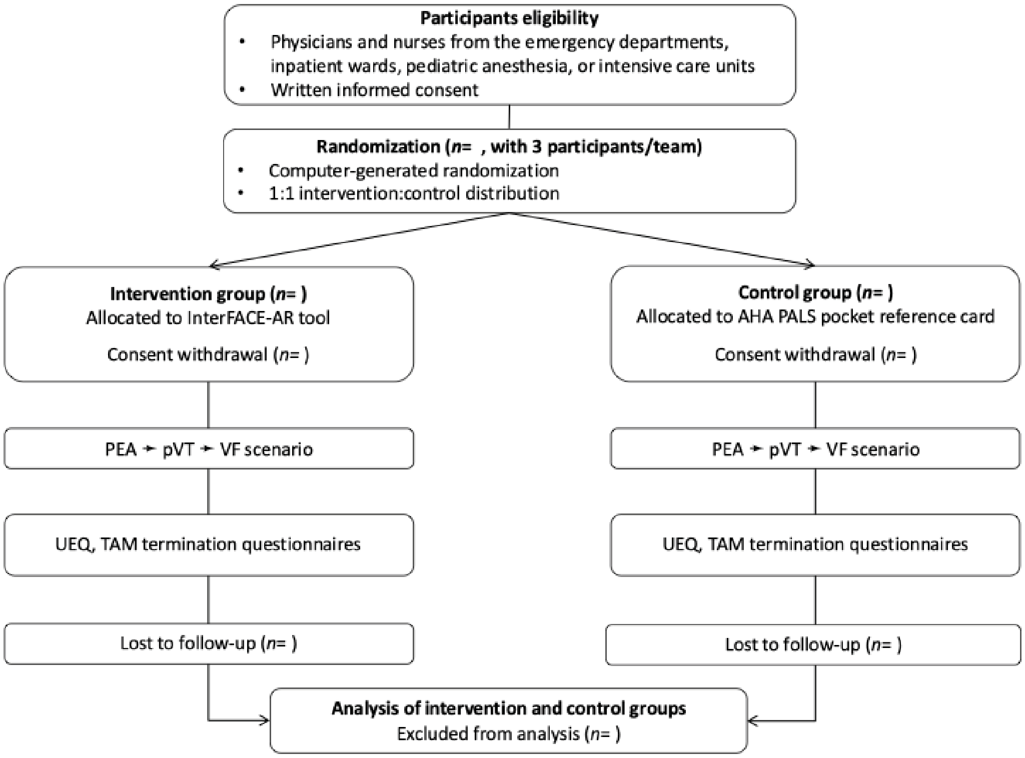

Figure 2. Trial flow chart.

PEA, pulseless electrical activity; pVT, pulseless ventricular tachycardia; TAM, technology acceptance model; UEQ, user experience questionnaire; VF, ventricular fibrillation.

2.5 Participants and Eligibility Criteria

Eligible team leaders will be attending physicians, fellows, senior residents, or physician assistants in emergency medicine, pediatric emergency medicine, general pediatrics, pediatric critical care, or pediatric anesthesia of both pediatric hospitals. Eligible medication and charting nurses will be nurses

in pediatric emergency medicine, general pediatrics, or pediatric intensive care units. All participants will be required to have completed prior basic life support training. Exclusion criteria: (1) previously participated in the design, development, or usability assessment of the system; or (2) unable to perform tasks required of the role.

2.6 Recruitment Process

To ensure sufficient subject enrolment for reaching the target sample size on the day of participation, teams will be randomly recruited and scheduled weeks prior to the study commencement by a blinded non-investigator. To mitigate preparation bias, they will be informed about the upcoming simulation study without knowledge of its objectives and outcomes. In cases of participant unavailability, the research team will arrange for a replacement.

2.7 Criteria for Discontinuing or Modifying Interventions

We do not anticipate modification or discontinuation of the allocated simulation condition for trial participants. Each session will follow the planned protocol for a full 60-minute scenario per participant, scheduled several weeks in advance to ensure availability of the participants without repercussions on their normal duty. All participants will have prior authorization from their hierarchy to attend. As a simulation study with high-fidelity manikins and no patient involvement, the intervention is highly feasible and carries minimal risk. Only in exceptional circumstances such as an unforeseen technical failure of the simulator/AR device or a participant-initiated request may the session be briefly paused or, if necessary, terminated and reported.

2.8 Strategies to Improve Adherence

We don’t anticipate any major issues with adherence based on our prior experience conducting simulation-based multicenter studies. We will implement a multifaceted approach to ensure compliance with our research protocol: (1) development of standard operating procedures and a research protocol guide; (2) submission of data from at least one pilot session per site, which will be reviewed for protocol adherence before recruitment; (3) monthly review of study data and videos for protocol adherence and quality control. Recruitment process to ensure adherence was described in section 2.4 above.

3. Trial Interventions

3.1 Interventions

The trial will assess the impact of the InterFACE-AR system on two interventions designed to provide decision support during pediatric in-hospital cardiac arrest:

- **Intervention A**: *InterFACE-AR System* – data collected from the Guiding-Pad app will be fed to the two AR headsets for the team leader and medication nurse to provide role-specific decision support, and to the TeamScreen, which will be mounted on a wall, in clear view for the entire resuscitation team to see.
- **Control group**: *AHA PALS Pocket Reference Cards* – all participants will have access to the AHA PALS pocket reference cards, which is the mostly commonly used cognitive aid during resuscitation.

3.2 Orientation and Simulation Scenarios

Following randomization (1:1 ratio), all participants will view a standardized orientation video describing the clinical environment, equipment, manikin functionality, and participant roles. The intervention group will receive additional orientation to the InterFACE-AR system, comprised of (1) a 10-minute instructional video describing the system's components and functions; (2) a 5-minute hands-on training session using the assigned device (Guiding Pad app for medication nurse, AR headsets for team leader and medication nurse) and comprised of opportunity to use and practice their assigned task, guided by a checklist, and (3) a 4-minute verbal scripted walk-through scenario allowing participants to use the system in their assigned roles.

A 12-minute CA simulation scenario (pulseless electrical activity [4 minutes] → pulseless ventricular tachycardia [4 minutes] → ventricular fibrillation [4 minutes] → return of spontaneous circulation) will be run by both groups. Both recruitment sites will utilize the identical pediatric manikin (SimJunior™, Laerdal Corporation), specifically designed and calibrated for CPR training[4]. All simulation scenarios will be tightly standardized by using a scenario template with highly scripted actor roles and patient progression. After the session, participants will be instructed not to disclose any information from the scenario to other participants to prevent any preparation bias.

4. Outcomes

The Primary outcome measure will by the time to first dose of epinephrine, defined as the interval in seconds from recognition of CA at the start of the scenario to completion of epinephrine injection**.**
Secondary outcome measures will be:

- Adherence to ALS guidelines: (i) Time to initiation of CPR; (ii) Time to defibrillation; (iii) Time to delivery of first and second drug to treat underlying condition; (iv) Time to secure definitive airway; (v) Medication errors (absolute count and frequency; assessed immediately post-scenario by measuring residual volumes in collected and labeled syringes), as described previously in one of our RCTs[24] as a deviation in drug dose exceeding 10% from the correct weight-based dose according to

predefined, expert consensus-based criteria[25] (vi) Frequency and duration for pauses in CPR (captured by video review, see below).

• CPR quality: (i) Chest compression fraction (CCF) (percentage of time performing CC during cardiac arrest); and (ii) Peri-shock pause durations.

• User experience: assessed using the User Experience Questionnaire (UEQ), a 26-item, 7-point scale questionnaire with supportive validity evidence, grouped into 6 scales covering efficiency, perspicuity dependability, attractiveness, stimulation, and novelty[26].

• Technology acceptance: measured with the Technology Acceptance Model (TAM) survey[27], evaluating perceived usefulness and ease of use.

5. Participant Timeline

The total study period to enrol all participants will take 1 month.

	STUDY PERIOD				
	Pre-enrolment	Enrolment	Pre-study baseline / allocation	12-min scenario	Close-out
TIMEPOINT	$-t_2$	$-t_1$	t_0	t_1	t_x
STUDY PROCEDURES					
Invitation to participate	✓				
Recruitment		✓			
Eligibility screening		✓			
Informed consent		✓			
InterFACE-AR orientation/practice		✓			
Randomization			✓		
INTERVENTION					
Intervention group (InterFACE-AR)				✓	
Control group (conventional)				✓	
ASSESSMENTS					
Demographics + clinical data (CRF)			✓		
Primary outcome					
Time to first dose of epinephrine				✓	
Secondary outcomes					
Time to initiation of CPR				✓	
Time to defibrillation				✓	
Time to delivery of other drugs				✓	
Time to definitive airway				✓	
Medication errors (video review)				✓	
CPR pauses: frequency/duration (video review)				✓	
CCF, peri- shock pause via Zoll R Series)				✓	
User Experience Questionnaire (UE, survey)					✓
Technology Acceptance Model (TAM, survey)					✓

Figure 3. Standard Protocol Items: Recommendations for Interventional Trials checklist (SPIRIT) figure.

6. Data Collection

6.1 Methods of Measurement

Times to critical interventions (in seconds), including initiation of CPR, time to medication and defibrillation orders, and definitive airway, will be abstracted from a standardized video setup. The scenario will be videotaped from a bird's-eye view angled at 20-30°, using a GoPro Hero 12 Black (San Mateo, CA, USA) mounted on a tripod positioned 7 feet from the foot of the bed and 6 feet 6 inches above the floor, recording in 4K resolution at 60 frames per second (16:9, wide-angle). The camera position and settings will be standardized across sites. Videos will be stored in duplicate on secure, password-protected hard drives in locked rooms at each study site. Adherence to ALS guidelines will be assessed by video review conducted independently by two trained, calibrated raters. Chest compression fraction (CCF) and peri-shock pause duration will be obtained directly from the Zoll R Series defibrillator. Following completion of the scenario, participants will fill out the User Experience Questionnaire[26], and TAM questionnaire[27] (see secondary outcomes below), all of which have been previously utilized in our previous studies. Compliance with AHA targets will follow published definitions[28-30]. Study data will be collected and managed in REDCap (Geneva University Hospitals), a secure, web-based platform with audit trails and validated data-capture features, allowing controlled export to statistical software.

6.2 Retention and Follow-up

All activities occur in a single, same-day session**. N**o post-session follow-up is planned. Retention is promoted by scheduling sessions several weeks in advance with managerial approval, issuing confirmation/reminder notices**,** and on-site check-in to minimize delays**. A**ttendance is logged, and brief rescheduling windows are available on the day if needed.

If a participant discontinues or deviates from the assigned condition, we will record: allocation, reason/timing of discontinuation/deviation, and all outcome data available up to that **point.** These data will be included in the intention-to-treat dataset. No additional outcome collection after the session will occur.

7. Statistical considerations

7.1 Power and Sample Size Calculation

The calculation is based on the primary outcome, time to first epinephrine dose. Prior data report a median of approximately 165 seconds (IQR of 139 to 173 seconds)[31]. Using the Greco et al. approach[32], and using the 1st and 3rd quartiles, we estimate SD as (173-139)/1.35 = 25.2 seconds. With a two-sided

α=.05 and 90% power to detect a clinically meaningful absolute reduction of 35 seconds, and assuming similar variability between groups, 18 teams (9 per arm) are required.

7.2 Group Allocation

Randomization will be conducted in a 1:1 ratio at the level of the team and stratified by study site using an online random number generator[33]. Randomization will be conducted in blocks of two to ensure equal distribution of teams across study arms. Study packages will be prepared with opaque envelopes to achieve allocation concealment. Sequentially numbered recruitment packages provided for each site will contain unique identifier codes for participants, along with study arm assignments. Personnel who enroll participants and those who assign participants to interventions will have no advance access to the sequence. On the trial day, the principal investigators or delegated investigators will obtain written informed consent from participants before any study procedures, and selection criteria will be checked prior to participation in the study. Immediately after written consent, the authorized staff will access only the assignment function to obtain the participant's allocation. Assignment will occur once per participant, with audit trails documenting day and time.

7.3 Blinding

Due to the nature of the intervention, blinding of reviewers to group allocation will not be feasible. Post-scenario video review will be done independently by two trained raters. In the case of disagreement, a third independent evaluator will help reach a consensus. An intraclass correlation coefficient will be computed. The investigators will remain unaware of the outcomes until all data are unlocked for analysis at the end of the trial.

7.4 Statistical Analysis Plan

7.4.1 Data Summary

We will summarize the demographic characteristics of participants in both the InterFACE-AR group and the PALS pocket card group. For numeric variables (e.g., height), the mean and standard deviation will be reported. For categorical variables (e.g., sex, profession), counts and percentages will be presented. A template for the demographic summary table is provided below.

	**Participants[a]**	
**Characteristics**	**InterFACE-AR (n = 27)**	**PALS pocket cards (n = 27)**
Sex		
Male	X (X%)	X (X%)
Female	X (X%)	X (X%)
Height (SD), cm	Mean (SD)	Mean (SD)
Profession		

Attending physician	X (X%)	X (X%)
Resident / Fellow	X (X%)	X (X%)
Physician assistant	X (X%)	X (X%)
Nurse	X (X%)	X (X%)
Nurse Practitioner	X (X%)	X (X%)
Resuscitation training (BLS/ACLS/PALS/PEARS)		
Instructor status	X (X%)	X (X%)
< 1	X (X%))	X (X%)
1 - 6	X (X%)	X (X%)
7 - 12	X (X%))	X (X%)
> 12	X (X%)	X (X%)
Resuscitation events involved in, n (SD)	Mean (SD)	Mean (SD)
Simulated resuscitation events involved in, n (SD)	Mean (SD)	Mean (SD)
Real CPR in the past year, n (SD)	Mean (SD)	Mean (SD)
Simulated CPR in the past year, n (SD)	Mean (SD)	Mean (SD)
Experience of headset with immersive technology		
Never	X (X%)	X (X%)
Once a month or less	X (X%)	X (X%)
Once a week to once a month	X (X%)	X (X%)
Experience of using AR for education		
Never	X (X%)	X (X%)
Once a month or less	X (X%)	X (X%)
Once a week to once a month	X (X%)	X (X%)

7.4.2 Primary Outcome - Time to administer 1st dose of epinephrine

Due to the small sample size, non-parametric approaches will be used to account for potential violations of assumptions underlying parametric tests. Boxplots will be generated to assess the distribution of data in both study arms. If the data are approximately symmetric and normally distributed, the mean difference will be estimated; if the data are skewed or non-normally distributed, the median difference will be estimated instead. Bootstrapping with 10,000 resamples will be used to calculate 95% confidence intervals for the estimated differences. Permutation tests with 10,000 iterations will be conducted to determine 2-sided p-values.

7.4.3 Secondary Outcomes – Time to other critical tasks during cardiac arrest management

These outcomes include time to initiate CPR, time to first dose of defibrillation, time to establish advanced airway, time to 1st and 2nd drugs to treat hyperkalemia, chest compression fraction and peri-shock durations. Time to these critical tasks and BLS outcomes (e.g., chest compression fraction, peri-shock pause durations) will be analyzed using the same non-parametric approaches applied to the primary outcomes. After assessing the data distribution, bootstrapping and permutation tests with 10,000

iterations will be used to estimate 95% confidence intervals and p-values for the differences. A template table is presented below.

**Variables**	**InterFACE-AR (n = 9)**	**PALS cards (n = 9)**	**Median/mean Difference (95% CI)***	***P* value****
Outcome 1	X (X – X)	X (X – X)	X (X, X)	X
Outcome 2	X (X – X)	X (X – X)	X (X, X)	X
…	X (X – X)	X (X – X)	X (X, X)	X

7.4.4 Secondary Outcomes – Medication dose deviations

Medication dose deviations are defined as 10% deviated from the guideline dosage. Epinephrine, amiodarone and calcium doses will be examined and measured as dichotomous outcomes. They will be presented as counts and percentages for each study arm. Odds ratios will be calculated to assess the relative effect of the intervention. To address small cell counts, the Haldane–Anscombe correction[34] will be applied. Risk differences (RD) will also be calculated to assess the absolute effect of the intervention, with 95% confidence intervals estimated using Newcombe's method[35]. Fisher's exact test will be used to calculate p-values. A template table is presented below.

	**InterFACE-AR**	**PALS cards**	**Odds ratio (95% CI)***	**Risk difference (95% CI)****	***P* value*****
Epinephrine dose deviation >10%, n (%)	X (X%)	X (X%)	X (X, X)	X (X, X)	X
Amiodarone dose deviation >10%, n (%)	X (X%)	X (X%)	X (X, X)	X (X, X)	X
Calcium dose deviation > 10%, n (%)	X (X%)	X (X%)	X (X, X)	X (X, X)	X

7.4.5 Secondary outcomes –Timing of subsequent epinephrine, pulse check and defibrillation

Timing of subsequent epinephrine, pulse check and defibrillation will be quantified as the deviation from the theoretic target intervals (theoretical epinephrine interval: every 4 minutes; and theoretical pulse check and defibrillation interval: every 2 minutes). Mixed effect linear regression models will be used to evaluate the effect of intervention on the absolute deviation from these theoretical value – treating early and delayed events equally (i.e., 10 seconds too early and 10 seconds too late are both considered 10-second deviation). To account for multiple measurements within the same simulation event, a random intercept for event will be included in the model. Model estimates will be reported as mean differences with corresponding 95%CI for all these outcomes.

7.4.6 Secondary outcomes - UEQ and TAM (for intervention group only)

The User Experience Questionnaire (UEQ) and the Technology Acceptance Model (TAM) survey will be administered to participants in the InterFACE-AR group only. Rather than presenting data for each individual item, we will report the statistical summaries (mean and standard deviation) for each construct within both tools. The UEQ includes six constructs: attractiveness, perspicuity, efficiency, dependability, stimulation, and novelty. The TAM includes two constructs: perceived usefulness and perceived ease of use. For each construct, the mean and standard deviation will be reported, and Cronbach's alpha will be calculated to assess the internal consistency of the items within each construct.

In the case of missing data, a complete case analysis will be conducted. No multiple imputations are planned. All statistical tests will be two-sided with a type one error risk of 5%. All statistical analyses will be performed with R software (version 4.5.0; Foundation for Statistical Computing).

8. Safety Assessments

8.1 Risks to Participants

The trial will be conducted under simulated conditions. There are no risks to the safety of participants. No safety parameters will be recorded nor analyzed.

8.2 Adverse Events and Serious Events

This simulation-only study will not prospectively collect or maintain an AE/SAE dataset. Given the minimal-risk, training-environment design with no patient involvement, formal AE/SAE reporting was not planned and will not be performed. Instead, for operational safety, study sites will manage any immediate participant concerns during sessions (e.g., discomfort, dizziness, motion-sickness with AR, equipment issues), pause or stop scenarios as needed, and note session interruptions, device malfunctions, or withdrawals in session/training logs.

8.3 Confidentiality

Confidentiality of study subjects will be ensured throughout the research process. All study data will be securely entered into the access-controlled and password protected REDCap data management system (REDCap, Vanderbilt University, Nashville, TN, USA; https://www.project-redcap.org/resources/citations/) hosted at Geneva University Hospitals. Each participant will be assigned an individual identifying code devoid of personal information. REDCap maintains a built-in audit trail that logs all user activity and all pages viewed by every user, including contextual information (e.g., records being accessed). Only anonymized data will be used for statistical analysis. No personal participant information will be disclosed outside of the study without explicit written consent. Individual

performance during the resuscitation scenario will be kept confidential and will not be shared at the institutional level.

9. Administrative Aspects, Monitoring and Publication

9.1 Handling and Storage of Data and Documents

This simulation-only study will generate video recordings of simulated IHCA sessions, session timings/performance metrics, and anonymized questionnaires (including training grade for stratification). Video files will be stored in duplicate on an institution-managed, access-controlled drive in a password-protected folder restricted to authorized study staff. Participants will be assigned a study code. Any linkage file (code–identity) will be stored separately with restrictive access. Questionnaires will be completed in REDCap without direct identifiers and accessed only via password. Video will be used solely for research metric extraction. The coded research dataset will be de-identified. No patient data are collected. The trial will comply with the Swiss Federal Act on Data Protection (FADP) and, where applicable, the EU GDPR, including data minimization, purpose limitation, and timely de-identification.

9.2 Trial Steering Committee

The trial steering committee, composed of Dr Johan Siebert and Dr Adam Cheng, will provide oversight of trial conduct, analysis, and reporting. The committee will meet weekly by teleconference to review progress, risks, and protocol adherence. A consolidated project plan (Gantt) will guide milestones and deliverables.

9.3 Monitoring and Quality Assurance

Day-to-day operations will be coordinated by an international research coordinator, with site research assistants responsible for local logistics, training, and data capture. Each site will submit a brief weekly progress report to the trial steering committee. The principal investigators and research coordinator will monitor data collection activities, and maintain data integrity and quality control. Data will be collected in REDCap and stored on a secure, password-protected institutional server at Geneva Children's Hospital, with access restricted to authorized study staff. Centralized data quality control will be performed weekly at the lead site, with feedback to sites and corrective actions as needed. Interpretation and publication of results will be the responsibility of the steering committee.

9.4 Interim Analysis

We will not perform interim analyses as our trial has a short duration and no potential serious outcomes. Our team has mitigation strategies in place to ensure minimal disruption, including: secured space within

simulation centers for recruitment, established protocols for recruiting participants, and a backup plan (i.e., redistribution of recruitment across sites) for recruiting participants should we fall behind schedule.

10. Ethics

10.1 Research Ethics Approval

Ethics approval was obtained at both sites (Switzerland and Canada).

10.1 Protocol Amendments

Important modifications, such as eligibility, outcomes, sample size, study procedures, will be submitted to the Ethics Committee for approval, communicated to all sites, and updated on the trial registry and in the protocol manual before implementation.

11. Relevance and Impact

We propose implementing an AR-based digital health solution at the point of care to address challenges in pediatric IHCA. This could serve as a valuable decision support tool, thus laying the foundation for improved, less stressful, and safer digitally-enabled care. InterFACE-AR anticipated impact spans both clinical practice and academic learning in ALS care.

11.1 Clinical practice

Improving adherence to ALS guidelines and ultimately increasing survival rates and long-term outcomes in a field where mortality or sequelae remain significant. By integrating real-time AHA PALS algorithms and role-specific information into the visual field of resuscitation teams via AR headsets and large screens, we address current gaps in current practices and pave the way for future research. With its technology-driven approach, our project holds the potential for cost-effective deployment globally, aligning with the World Health Organization Global Strategy on Digital Health[36].

11.2 Academic learning

Through the integration of large screens and AR technology into educational programs, our project will provide practical experience with cutting-edge technologies for simulation-based training, ensuring better preparation to effectively apply theoretical knowledge in real-world settings. Additionally, our project could create opportunities for interdisciplinary collaboration and knowledge sharing, for instance with adult resuscitation, facilitating a richer and more immersive learning experience for students and clinicians.

12. References

1. Cheng A, Hunt EA, Donoghue A, et al. Examining pediatric resuscitation education using simulation and scripted debriefing: a multicenter randomized trial. *JAMA Pediatr*. Jun 2013;167(6):528-36. doi:10.1001/jamapediatrics.2013.1389
2. Cheng A, Rodgers DL, van der Jagt E, Eppich W, O'Donnell J. Evolution of the Pediatric Advanced Life Support course: enhanced learning with a new debriefing tool and Web-based module for Pediatric Advanced Life Support instructors. *Pediatr Crit Care Med*. Sep 2012;13(5):589-95. doi:10.1097/PCC.0b013e3182417709
3. Anderson R, Sebaldt A, Lin Y, Cheng A. Optimal training frequency for acquisition and retention of high-quality CPR skills: A randomized trial. *Resuscitation*. Feb 2019;135:153-161. doi:10.1016/j.resuscitation.2018.10.033
4. Cheng A, Brown LL, Duff JP, et al. Improving cardiopulmonary resuscitation with a CPR feedback device and refresher simulations (CPR CARES Study): a randomized clinical trial. *JAMA Pediatr*. Feb 2015;169(2):137-44. doi:10.1001/jamapediatrics.2014.2616
5. Lin Y, Cheng A, Grant VJ, Currie GR, Hecker KG. Improving CPR quality with distributed practice and real-time feedback in pediatric healthcare providers - A randomized controlled trial. *Resuscitation*. Sep 2018;130:6-12. doi:10.1016/j.resuscitation.2018.06.025
6. Cheng A, Duff JP, Kessler D, et al. Optimizing CPR performance with CPR coaching for pediatric cardiac arrest: A randomized simulation-based clinical trial. *Resuscitation*. Nov 2018;132:33-40. doi:10.1016/j.resuscitation.2018.08.021
7. Cheng A, Kessler D, Lin Y, et al. Influence of cardiopulmonary resuscitation coaching and provider role on perception of cardiopulmonary resuscitation quality during simulated pediatric cardiac arrest. *Pediatr Crit Care Med*. Apr 2019;20(4):e191-e198. doi:10.1097/PCC.0000000000001871
8. Hunt EA, Jeffers J, McNamara L, et al. Improved Cardiopulmonary Resuscitation Performance With CODE ACES(2): A Resuscitation Quality Bundle. *J Am Heart Assoc*. Dec 18 2018;7(24):e009860. doi:10.1161/JAHA.118.009860
9. Kuyt K, Park SH, Chang TP, Jung T, MacKinnon R. The use of virtual reality and augmented reality to enhance cardio-pulmonary resuscitation: a scoping review. *Adv Simul (Lond)*. Apr 12 2021;6(1):11. doi:10.1186/s41077-021-00158-0
10. Siebert JN, Ehrler F, Gervaix A, et al. Adherence to AHA guidelines when adapted for augmented reality glasses for assisted pediatric cardiopulmonary resuscitation: a randomized controlled trial. *J Med Internet Res*. May 29 2017;19(5):e183. doi:10.2196/jmir.7379
11. Ehrler F, Siebert J, Haddad K, et al. Adapting Guidelines for Google Glass: the Case of Pediatric CPR. *Stud Health Technol Inform*. 2016;224:141-5.
12. Siebert JN, Lacroix L, Cantais A, Manzano S, Ehrler F. The impact of a tablet app on adherence to American Heart Association guidelines during simulated pediatric cardiopulmonary resuscitation: randomized controlled trial. *J Med Internet Res*. May 27 2020;22(5):e17792. doi:10.2196/17792
13. Weyhenmeyer JA, Peterson ED, Beam C, et al. American Heart Association Focusing Research Rigor on Digital Health. *J Am Heart Assoc*. Jan 16 2024;13(2):e032870. doi:10.1161/JAHA.123.032870
14. Schmucker M, Haag M. Automated Size Recognition in Pediatric Emergencies Using Machine Learning and Augmented Reality: Within-Group Comparative Study. *JMIR Form Res*. Sep 20 2021;5(9):e28345. doi:10.2196/28345
15. Gsaxner C, Li J, Pepe A, et al. The HoloLens in medicine: A systematic review and taxonomy. *Med Image Anal*. Apr 2023;85:102757. doi:10.1016/j.media.2023.102757
16. Cheng A, Auerbach M, Hunt EA, et al. Designing and conducting simulation-based research. *Pediatrics*. Jun 2014;133(6):1091-101. doi:10.1542/peds.2013-3267

17. Adler MD, Overly FL, Nadkarni VM, et al. An Approach to Confederate Training Within the Context of Simulation-Based Research. *Simul Healthc*. Oct 2016;11(5):357-362. doi:10.1097/SIH.0000000000000172
18. Cheng A, Kessler D, Mackinnon R, et al. Reporting guidelines for health care simulation research: extensions to the CONSORT and STROBE statements. *Simul Healthc*. Aug 2016;11(4):238-48. doi:10.1097/SIH.0000000000000150
19. Eysenbach G, Consort-Ehealth Group. CONSORT-EHEALTH: improving and standardizing evaluation reports of Web-based and mobile health interventions. *J Med Internet Res*. Dec 31 2011;13(4):e126. doi:10.2196/jmir.1923
20. World Medical Association. World Medical Association Declaration of Helsinki: ethical principles for medical research involving human subjects. *JAMA*. Nov 27 2013;310(20):2191-4. doi:10.1001/jama.2013.281053
21. ICH Harmonised Tripartite Guideline. Statistical principles for clinical trials. International Conference on Harmonisation E9 Expert Working Group. *Stat Med*. Aug 15 1999;18(15):1905-42.
22. Hopewell S, Chan AW, Collins GS, et al. CONSORT 2025 statement: updated guideline for reporting randomised trials. *BMJ*. Apr 14 2025;389:e081123. doi:10.1136/bmj-2024-081123
23. Chan AW, Boutron I, Hopewell S, et al. SPIRIT 2025 statement: updated guideline for protocols of randomised trials. *BMJ*. Apr 28 2025;389:e081477. doi:10.1136/bmj-2024-081477
24. Siebert JN, Bloudeau L, Combescure C, et al. Effect of a mobile app on prehospital medication errors during simulated pediatric resuscitation: a randomized clinical trial. *JAMA Netw Open*. Aug 2 2021;4(8):e2123007. doi:10.1001/jamanetworkopen.2021.23007
25. Roumeliotis N, Pullenayegum E, Rochon P, Taddio A, Parshuram C. A modified Delphi to define drug dosing errors in pediatric critical care. *BMC Pediatr*. Oct 21 2020;20(1):488. doi:10.1186/s12887-020-02384-3
26. Schrepp M, Thomaschewski J, Hinderks A. Construction of a benchmark for the user experience questionnaire (UEQ). 2017;
27. King WR, He J. A meta-analysis of the technology acceptance model. *Information & management*. 2006;43(6):740-755.
28. Merchant RM, Topjian AA, Panchal AR, et al. Part 1: Executive Summary: 2020 American Heart Association Guidelines for Cardiopulmonary Resuscitation and Emergency Cardiovascular Care. *Circulation*. Oct 20 2020;142(16_suppl_2):S337-S357. doi:10.1161/CIR.0000000000000918
29. Panchal AR, Bartos JA, Cabanas JG, et al. Part 3: Adult Basic and Advanced Life Support: 2020 American Heart Association Guidelines for Cardiopulmonary Resuscitation and Emergency Cardiovascular Care. *Circulation*. Oct 20 2020;142(16_suppl_2):S366-S468. doi:10.1161/CIR.0000000000000916
30. Topjian AA, Raymond TT, Atkins D, et al. Part 4: pediatric basic and advanced life support: 2020 American Heart Association guidelines for cardiopulmonary resuscitation and emergency cardiovascular care. *Circulation*. Oct 20 2020;142(16_suppl_2):S469-S523. doi:10.1161/CIR.0000000000000901
31. Corazza F, Snijders D, Arpone M, et al. Development and usability of a novel interactive tablet app (PediAppRREST) to support the management of pediatric cardiac arrest: pilot high-fidelity simulation-based study. *JMIR Mhealth Uhealth*. Oct 1 2020;8(10):e19070. doi:10.2196/19070
32. Greco T, Biondi-Zoccai G, Gemma M, Guérin C, Zangrillo A, Landoni G. How to impute study-specific standard deviations in meta-analyses of skewed continuous endpoints? *World Journal of Meta-Analysis*. 2015;3(5):215-224.

33. Sealed Envelope. Simple randomisation service. Accessed 13 June, 2025. https://www.sealedenvelope.com
34. Anscombe FJ. On estimating binomial response relations. *Biometrika*. 1956;43(3/4):461-464.
35. Newcombe RG. Interval estimation for the difference between independent proportions: comparison of eleven methods. *Statistics in medicine*. 1998;17(8):873-890.
36. Organization WH. Global strategy on digital health 2020–2025. 2021. *License: CC BY-NC-SA*. 2021;3

SPIRIT 2025 checklist of items to address in a randomized trial protocol

Section / Topic	No	SPIRIT 2025 checklist item description	Reported on page no.
**Administrative information**			
Title and structured summary	1a	Title stating the trial design, population, and interventions, with identification as a protocol	1
	1b	Structured summary of trial design and methods, including items from the World Health Organization Trial Registration Data Set	4
Protocol version	2	Version date and identifier	1
Roles and responsibilities	3a	Names, affiliations, and roles of protocol contributors	2
	3b	Name and contact information for the trial sponsor	2
	3c	Role of trial sponsor and funders in design, conduct, analysis, and reporting of trial; including any authority over these activities	2
	3d	Composition, roles, and responsibilities of the coordinating site, steering committee, endpoint adjudication committee, data management team, and other individuals or groups overseeing the trial, if applicable	2
**Open science**			
Trial registration	4	Name of trial registry, identifying number (with URL), and date of registration. If not yet registered, name of intended registry	1
Protocol and statistical analysis plan	5	Where the trial protocol and statistical analysis plan can be accessed	With the main article, as a supplemental material
Data sharing	6	Where and how the individual de-identified participant data (including data dictionary), statistical code, and any other materials will be accessible	With the main article, as a supplemental material
Funding and conflicts of interest	7a	Sources of funding and other support (e.g., supply of drugs)	2
	7b	Financial and other conflicts of interest for principal investigators and steering committee members	2
Dissemination policy	8	Plans to communicate trial results to participants, healthcare professionals, the public, and other relevant groups (e.g., reporting in trial registry, plain language summary, publication)	8
**Introduction**			
Background and rationale	9a	Scientific background and rationale, including summary of relevant studies (published and unpublished) examining benefits and harms for each intervention	5-6
	9b	Explanation for choice of comparator	5-6

Objectives	10	Specific objectives related to benefits and harms	6
**Methods: Patient and public involvement, trial design**			
Patient and public involvement	11	Details of, or plans for, patient or public involvement in the design, conduct, and reporting of the trial	8
Trial design	12	Description of trial design including type of trial (e.g., parallel group, crossover), allocation ratio, and framework (e.g., superiority, equivalence, non-inferiority, exploratory)	7-8
**Methods: Participants, interventions, and outcomes**			
Trial setting	13	Settings (e.g., community, hospital) and locations (e.g., countries, sites) where the trial will be conducted	7-8
Eligibility criteria	14a	Eligibility criteria for participants	8-9
	14b	If applicable, eligibility criteria for sites and for individuals who will deliver the interventions (e.g., surgeons, physiotherapists)	NA
Intervention and comparator	15a	Intervention and comparator with sufficient details to allow replication including how, when, and by whom they will be administered. If relevant, where additional materials describing the intervention and comparator (e.g., intervention manual) can be accessed	9-10
	15b	Criteria for discontinuing or modifying allocated intervention/comparator for a trial participant (e.g., drug dose change in response to harms, participant request, or improving/worsening disease)	9
	15c	Strategies to improve adherence to intervention/comparator protocols, if applicable, and any procedures for monitoring adherence (e.g., drug tablet return, sessions attended)	9
	15d	Concomitant care that is permitted or prohibited during the trial	NA
Outcomes	16	Primary and secondary outcomes, including the specific measurement variable (e.g., systolic blood pressure), analysis metric (e.g., change from baseline, final value, time to event), method of aggregation (e.g., median, proportion), and time point for each outcome	10
Harms	17	How harms are defined and will be assessed (e.g., systematically, non-systematically)	NA
Participant timeline	18	Time schedule of enrollment, interventions (including any run-ins and washouts), assessments, and visits for participants. A schematic diagram is highly recommended (see Figure)	11
Sample size	19	How sample size was determined, including all assumptions supporting the sample size calculation	12-13
Recruitment	20	Strategies for achieving adequate participant enrollment to reach target sample size	9
**Methods: Assignment of interventions**			
Randomization:			
Sequence generation	21a	Who will generate the random allocation sequence and the method used	13
	21b	Type of randomization (simple or restricted) and details of any factors for stratification. To reduce predictability of a random sequence, other details of any planned restriction (e.g.,	13

		blocking) should be provided in a separate document that is unavailable to those who enroll participants or assign interventions	
Allocation concealment mechanism	22	Mechanism used to implement the random allocation sequence (e.g., central computer/telephone; sequentially numbered, opaque, sealed containers), describing any steps to conceal the sequence until interventions are assigned	13
Implementation	23	Whether the personnel who will enroll and those who will assign participants to the interventions will have access to the random allocation sequence	13
Blinding	24a	Who will be blinded after assignment to interventions (e.g., participants, care providers, outcome assessors, data analysts)	13
	24b	If blinded, how blinding will be achieved and description of the similarity of interventions	NA
	24c	If blinded, circumstances under which unblinding is permissible, and procedure for revealing a participant's allocated intervention during the trial	NA
**Methods: Data collection, management, and analysis**			
Data collection methods	25a	Plans for assessment and collection of trial data, including any related processes to promote data quality (e.g., duplicate measurements, training of assessors) and a description of trial instruments (e.g., questionnaires, laboratory tests) along with their reliability and validity, if known. Reference to where data collection forms can be accessed, if not in the protocol	12, 17
	25b	Plans to promote participant retention and complete follow-up, including list of any outcome data to be collected for participants who discontinue or deviate from intervention protocols	12
Data management	26	Plans for data entry, coding, security, and storage, including any related processes to promote data quality (e.g., double data entry; range checks for data values). Reference to where details of data management procedures can be accessed, if not in the protocol	17
Statistical methods	27a	Statistical methods used to compare groups for primary and secondary outcomes, including harms	12-16
	27b	Definition of who will be included in each analysis (e.g., all randomized participants), and in which group	12-16
	27c	How missing data will be handled in the analysis	16
	27d	Methods for any additional analyses (e.g., subgroup and sensitivity analyses)	NA
**Methods: Monitoring**			
Data monitoring committee	28a	Composition of data monitoring committee (DMC); summary of its role and reporting structure; statement of whether it is independent from the sponsor and funder; conflicts of interest and reference to where further details about its charter can be found, if not in the protocol. Alternatively, an explanation of why a DMC is not needed	17
	28b	Explanation of any interim analyses and stopping guidelines, including who will have access to these interim results and make the final decision to terminate the trial	17-18
Trial monitoring	29	Frequency and procedures for monitoring trial conduct. If there is no monitoring, give explanation	17-18
**Ethics**			
Research ethics approval	30	Plans for seeking research ethics committee/institutional review board approval	18

Protocol amendments	31	Plans for communicating important protocol modifications to relevant parties	18
Consent or assent	32a	Who will obtain informed consent or assent from potential trial participants or authorized proxies, and how	13
	32b	Additional consent provisions for collection and use of participant data and biological specimens in ancillary studies, if applicable	NA
Confidentiality	33	How personal information about potential and enrolled participants will be collected, shared, and maintained in order to protect confidentiality before, during, and after the trial	16
Ancillary and post-trial care	34	Provisions, if any, for ancillary and post-trial care, and for compensation to those who suffer harm from trial participation	NA
