# Supplementary material for: Augmented Reality–Guided Decision Support in Simulated Pediatric Cardiac Arrest: A Randomized Clinical Trial
Source: JAMA Netw Open. 2026 May 22;9(5):e2614030. doi: 10.1001/jamanetworkopen.2026.14030 (PMC13197869; doi:10.1001/jamanetworkopen.2026.14030)
Supplement: Supplement 2. — eAppendix 1. Recruitment and team assembly eAppendix 2. The InterFACE-AR decision support system eAppendix 3. Scenario procedures, actor roles and simulation environment eAppendix 4. Cardiac arrest simulation scenario eAppendix 5. Data collection and sources eFigure 1. Timing of critical resuscitation actions relative to AHA PALS targets eFigure 2. Timing of sequential epinephrine doses and deviations from AHA recommended intervals eFigure 3. Time to first and subsequent defibrillations, by study groups eFigure 4. Timing of sequential defibrillations and deviations from AHA recommended intervals eTable 1. Medication dosing accuracy, by study group eTable 2. User experience questionnaire eTable 3. Technology acceptance model eReferences [file jamanetwopen-e2614030-s002.pdf]

Supplemental Online Content

Siebert JN, Cheng A, De Masi A, et al. Augmented reality-guided decision support in simulated pediatric cardiac arrest: a randomized clinical trial. *JAMA Netw. Open.* 2026;9(5):e2614030. doi:10.1001/jamanetworkopen.2026.14030

eAppendix 1. Recruitment and team assembly

eAppendix 2. The InterFACE-AR decision support system

eAppendix 3. Scenario procedures, actor roles and simulation environment

eAppendix 4. Cardiac arrest simulation scenario

eAppendix 5. Data collection and sources

eFigure 1. Timing of critical resuscitation actions relative to AHA PALS targets

eFigure 2. Timing of sequential epinephrine doses and deviations from AHA recommended intervals

eFigure 3. Time to first and subsequent defibrillations, by study groups

eFigure 4. Timing of sequential defibrillations and deviations from AHA recommended intervals

eTable 1. Medication dosing accuracy, by study group

eTable 2. User experience questionnaire

eTable 3. Technology acceptance model

eReferences

This supplemental material has been provided by the authors to give readers additional information about their work.

© 2026 Siebert JN et al. *JAMA Network Open.*

eAppendix 1

Recruitment and team assembly

Teams consisted of three study participants (team leader, charting nurse, and medication nurse) and four trained research actors (two alternating as CPR provider/coach, one bedside nurse, and one as airway provider) to form a seven-person resuscitation team. They were assembled before randomization by a site coordinator who was not involved in data collection or analysis and did not have access to the allocation sequence. Teams were formed based on role availability and clinical schedules, using a prespecified approach designed to reflect usual ad hoc interprofessional resuscitation teams. Because coordinators worked within participating departments, they may have had routine professional familiarity with some clinicians. However, team assignment was based solely on availability and role requirements, rather than prior performance, experience, or interpersonal relationships. Participants were not informed of their teammates in advance and had no opportunity to prepare as a team, consistent with real-world IHCA conditions. Participants were informed about participation in a pediatric resuscitation simulation without disclosure of study hypotheses or outcome definitions.

© 2026 Siebert JN et al. *JAMA Network Open.*

eAppendix 2

The InterFACE-AR Decision Support System

Expanding on our previous prototype[1, 2], we redesigned the system using a user-centered, iterative process informed by clinical guidelines, observations and expert consensus. The InterFACE-AR (Interconnected and Focused mobile Applications in the patient Care Environment with Augmented Reality-based guidance) system is a team-based, multimodal decision-support platform designed to support pediatric resuscitation by delivering time-linked, role-specific guidance during cardiac arrest scenarios. The final InterFACE-AR platform (**eFigure1**) integrates three synchronized components. First, a tablet-based mobile app (Guiding Pad), operated by the medication nurse for real-time charting, displays the appropriate resuscitation algorithm and clinical status (e.g., rhythm phase, elapsed time, completed tasks, and timestamps of key interventions)[3]. These inputs drive the progression of the algorithm and synchronize information across system components. Second, clinical data are broadcast to a large shared display (TeamScreen) presenting the resuscitation team a continuously updated overview of the current algorithm, clinical status, including cardiac rhythm phase, elapsed time, task progression (completed, current, and upcoming tasks), and timestamps of key interventions[4]. The TeamScreen supports shared situational awareness across the team. Third, two AR headsets (Microsoft HoloLens 2™) deliver simultaneously role-specific, step-by-step, real-time guidance aligned with resuscitation algorithms to the team leader and medication nurse. The team leader interface provides algorithm navigation and prompts for key interventions (e.g., defibrillation, rhythm checks, and drug administration), whereas the medication nurse interface provides medication-specific guidance, including drug selection, dosing, and timing[5]. Time-linked prompts, including a fixed 4-minute timer for epinephrine within the recommended 3–5-minute interval, are integrated to support adherence to guidelines. All components are synchronized in real time, ensuring that data entered through the Guiding Pad are reflected across the TeamScreen and AR interfaces, thereby supporting coordinated team performance.

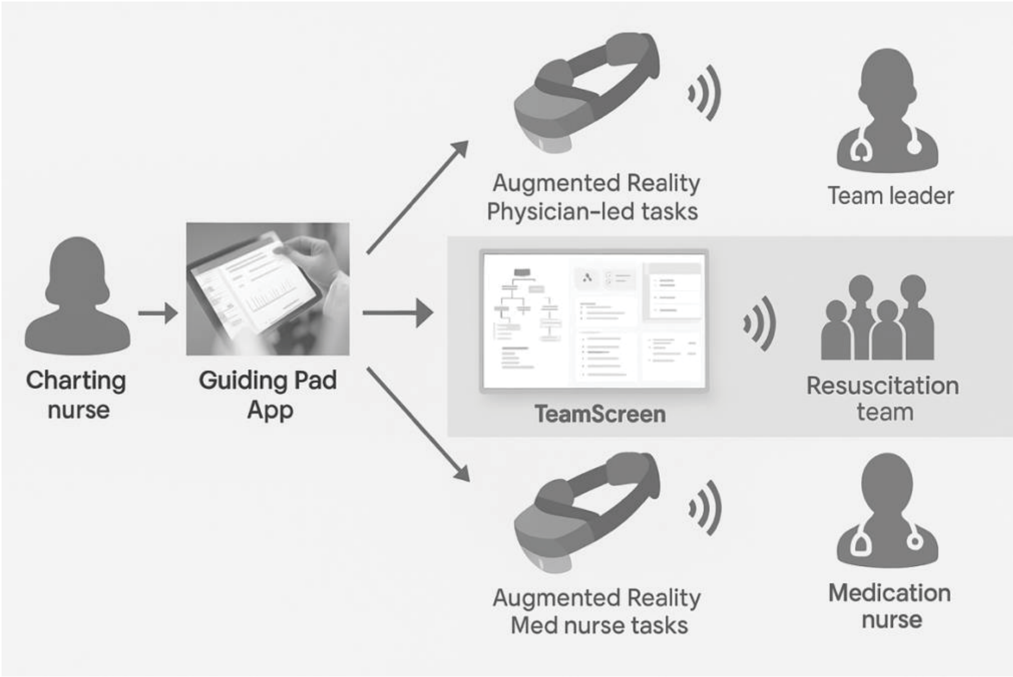

Structure and information flow of the InterFACE-AR system. The charting nurse enters patient data and completed tasks into the Guiding-Pad app on a tablet. This information is transmitted in real time via WiFi to three components: (1) an AR headset worn by the team leader, displaying role-specific prompts and guidance; (2) the TeamScreen, a large shared display visible to the resuscitation team, presenting the AHA PALS algorithms, patient data, and current and upcoming tasks; and (3) a second AR headset worn by the medication nurse, providing real-time guidance for medication preparation and administration.

© 2026 Siebert JN et al. *JAMA Network Open.*

eAppendix 3

Scenario procedures, actor roles and simulation environment

After allocation, all participants viewed a 3-minute standardized orientation video describing the clinical environment, equipment, manikin functionality, and roles. Both groups participated in an identical 4-minute verbal scripted walk-through scenario to rehearse their assigned roles within their allocated condition. The intervention group additionally received brief device-specific familiarization to ensure minimal operational proficiency with the AR system, comprised of (1) a 10-minute instructional video describing the system's components and functions; (2) a 5-minute hands-on training session using the assigned device. This additional familiarization was intended to support device use and not to provide additional clinical or teamwork training beyond what was provided to both groups. In lieu of InterFACE-AR training, participants assigned to the control group had opportunity to review the AHA PALS pocket reference card for 5 minutes.

Before each scenario, AR holograms were spatially calibrated and anchored within the physical room space using a standardized procedure across both study sites. The team leader's position and TeamScreen location were marked to ensure consistent room setup. Teams then managed a 12-minute standardized pediatric IHCA simulation scenario following a scripted template with predefined actor roles and patient progression, either with the support of InterFACE-AR or the AHA PALS pocket card (**Figure 1** in main manuscript). The scenario progressed through pulseless electrical activity (minutes 0-4), pulseless ventricular tachycardia (minutes 4-8), and ventricular fibrillation (minutes 8-12), ending with return of spontaneous circulation (**eAppendix 4**). The timing of rhythm transitions was fixed and identical across all teams, independent of CPR cycle timing, ensuring comparability between groups. Hyperkalemia as the underlying cause of CA was disclosed at 6 minutes and 30 seconds. All simulations used the same manikin (SimJuniorTM, Laerdal Corporation)47 and defibrillator (R Series, Zoll Medical Corporation). Teams were instructed to manage the scenario per AHA PALS guidelines, including drug preparation and administration, defibrillation, and addressing reversible causes. Full details of the scenario and data collection and sources are provided in the **eAppendices 4 and 5** below.

eAppendix 4

Cardiac arrest simulation scenario

Notes for Simulation Facilitator

All participants should be familiar with the simulator, allow time for them to feel pulses, auscultate chest, etc.

In order to standardize the orientation process, have participants view orientation video provided.

Begin with the manikin under a sheet
(Explain to participants removing the sheet will represent the patient arriving)

Please read this:
"*You are in the Pediatric Emergency Department and just received a call from pre-hospital providers. They are transporting an 8-year-old male with a 1-week history of bloody diarrhea, abdominal pain and decreased urine output at home. Upon arrival at their home, the child was poorly responsive with intermittent respirations and a weak pulse. They have established an IV, and they have been bagging him during transport. Parents gave an estimated weight of 25kg. They are just above to arrive. Estimated time of arrival is 15 seconds*"

After 15 seconds, remove the sheet and state:
"*The patient has arrived, prehospital personnel have left, they tell you parents are en route and the patient has no known past medical history. There is a functioning IV in the left antecubital fossa.*"

TURN ON ZOLL DEFIBRILLATOR at START of scenario (*see next page*)

© 2026 Siebert JN et al. *JAMA Network Open.*

Scenario Stage	Patient condition	Simulator Parameters	Expected Intervention	Confederate Roles/other notes
**PEA**<br>**4 min**	**History**<br>• 8-year-old boy<br>• Bloody diarrhea, abdominal pain, poorly responsive<br>• PMH: unknown<br><br>**Condition:**<br>• Unconscious, apneic<br><br>**Physical Exam**<br>• Monitor: PEA<br>• CNS: unconscious, eyes closed<br>• CVS: No Pulses Palpable, CRT 8 sec, mottled, no enlarged liver<br>• Resp: no spontaneous resp, clear breath sounds with ventilation	**Vitals**<br>• T 36.5<br>• HR 40<br>• RR 0<br>• SpO_2 N/A<br>• BP N/A<br>• $EtCO_2$ 15<br><br>**Condition**<br>• No Pulse<br><br>**Rhythm**<br>• PEA rate of 40 bpm	**Airway**<br>• Airway patent<br><br>**Breathing**<br>• BVM ventilation<br>• Monitor SpO2, EtCO2<br><br>**Circulation**<br>• Pulse check<br>• Second IV/IO<br>• Directs continuation of CPR<br>• Reassesses rhythm<br>• Recognizes and verbalizes PEA<br>• Directs immediately resumption of CPR<br>• Prepare and deliver epinephrine 0.25mg IV/IO	**Moulage**<br>• Dress in shorts/shoes<br>• 20G IV in right AC with connector<br><br>**Airway Provider will**<br>• Assess airway<br>• Initiate BVM ventilation<br>• Not offer information about vital signs or change in rhythm<br>• Not perform Chest compressions if asked<br><br>**Bedside Provider will**<br>Assess patient<br>Push epinephrine if asked<br>Not perform chest compressions if asked
**pVT**<br>**4 min**	**Condition:**<br>• Unconscious, apneic<br><br>**Physical Exam**<br>• T 36.5, HR 180, RR 0, SpO2 N/A<br>• Monitor: VT<br>• CNS: unconscious, eyes closed<br>• CVS: No Pulses Palpable, CRT 8 sec, mottled, no enlarged liver<br>• Resp: clear breath sounds with ventilation, easy to bag	**Vitals**<br>• T 36.5°C<br>• HR 180<br>• RR 0<br>• SpO_2 N/A<br>• BP N/A<br>• $EtCO_2$ 15 (if monitor attached)<br><br>**Condition**<br>• No Pulse<br>• CRT 8sec<br><br>**Rhythm**<br>• VT	**Airway**<br>• Secure definitive airway / consider intubation or LMA insertion<br><br>**Breathing**<br>• Start bagging patient immediately<br>• Check SpO_2<br><br>**Circulation**<br>• Pulse check<br>• Directs continuation of CPR<br>• Apply leads and turn on monitor<br>• Recognize and verbalize Pulseless VT<br>• Draw CBG and electrolytes/Ca-Mg/Cr<br>• 1st defibrillation at 2J/kg<br>• Directs immediate resumption of CPR<br>• 2nd defibrillation at 4J/kg<br>• Direct immediate resumption of CPR<br>• Prepare and deliver epinephrine 0.25mg IV<br>• Prepare amiodarone 125mg OR lidocaine 25 mg<br>• Search for and treat H's and T's<br>• Prompt to order CBG if not ordered yet (by 6 minutes)<br>• Search for and treat H's and T's	**Moulage**<br>• Dress in shorts/shoes<br>• 20G IV in right AC with connector<br><br>**Airway Provider will**<br>• Continue BVM ventilation<br>• Intubate patient when directed<br>• Not suggest when to secure definitive airway<br>• Not offer information about vital signs or change in rhythm<br>• Not perform Chest compressions if asked<br><br>**Bedside provider will**<br>• Assess patient<br>• Draw labs if asked<br>• Push epinephrine if asked<br>• Not perform chest compressions if asked<br><br>**Labs**<br>6.90/80/35/4/-21<br>Na^+ 131<br>K^+ 9.5<br>Cl^- 105<br>Cr 200<br>Glc 4.5<br><br>**Give lab results at 6:30 min**

© 2026 Siebert JN et al. *JAMA Network Open.*

**VF & HyperK 4 min**	**Condition:**<br>• Unconscious, apneic<br><br>Physical Exam<br>• Monitor: VF<br>• CNS: unconscious, eyes closed<br>• CVS: No Pulses Palpable, CRT 8 sec, mottled, no enlarged liver<br>• Resp: no spontaneous resp, clear breath sounds with ventilation	**Vitals**<br>• T 36.5°C<br>• HR 0<br>• RR 0<br>• SpO_2 N/A<br>• BP N/A<br>• $EtCO_2$ 15<br><br>**Condition**<br>• No Pulse<br><br>**Rhythm**<br>• VF	**Airway**<br>• Intubated patient (or LMA)<br><br>**Breathing**<br>• Continue bagging patient<br><br>**Circulation**<br>• Pulse check<br>• Directs continuation of CPR<br>• Reassesses rhythm<br>• Recognizes and verbalizes VF<br>• defibrillation at 4J/kg<br>• Directs immediately resumption of CPR<br>• Prepare and deliver of epinephrine 0.25mg IV<br>• Recognizes cause (hyperkalemia)<br>• Orders additional labs (Calcium, Mg, Bun, Creat, CK)<br><br>**Management of hyperkalemia**<br>• Calcium gluconate 10% solution or<br>Ca Chloride 10%<br>• Insulin/Glucose IV<br>• Salbutamol / Ventolin inhaled<br>• NaHCO3 IV<br>• Consider hemodialysis call nephrology/PICU	**Airway Provider will**<br>• Continue BVM ventilation<br>• Intubate patient or insert LMA when directed<br>• Not suggest when to secure definitive airway<br>• Not offer information about vital signs or change in rhythm<br>• Not perform Chest compressions if asked<br><br>**Bedside Provider will**<br>• Assess patient<br>• Draw labs if asked<br>• Push epinephrine and other medications if asked<br>• Not perform chest compressions if asked

After 12 minutes Facilitator will state: “*There is now a pulse, we are going to stop the simulation here, thank you*.”

eAppendix 5

Data Collection and Sources

Scenarios were videotaped from a bird's-eye view angled at 20-30°, using a GoPro Hero 12 Black (San Mateo, CA, USA) mounted on a tripod positioned 7 feet from the foot of the bed and 6 feet 6 inches above the floor, recording in 4K resolution at 60 frames per second (16:9, wide-angle). Videos were independently reviewed by two trained raters, one pediatric emergency physician and one pediatric emergency nurse, to capture time to initiation of CPR, time to first defibrillation, medications ordered (drug, dose, timing), and time to definitive airway. Two study videos were reviewed in duplicate. Interrater agreement for the primary outcome was excellent, with a Kappa coefficient of 0.9 for epinephrine administration. Interrater reliability for secondary outcomes was high, demonstrating an intraclass correlation coefficient of 0.9. The remaining study videos were rated independently. Due to structural and technical requirements, study participants, video analysists and statisticians were not blinded to group allocation, but the investigators remained unaware of the outcomes until all data were unlocked for analysis at the end of the trial.

Drugs drawn by the medication nurse were handed to a bedside actor, who verbally indicated administration without pushing the fluid. Labeled syringes were collected in a tray for post-scenario measurement of medication volumes. Chest compression fraction (CCF) and peri-shock pause duration were obtained directly from the Zoll R Series defibrillator. Following scenario completion, participants in the intervention group completed the 26-item User Experience Questionnaire (UEQ)[6], which rates six usability and affective constructs using a 7-point Likert scale from -3 (extremely bad) to +3 (extremely good, and 0 = neutral), and the Technology Acceptance Model (TAM) survey[7], which uses 7-point scale (1, strongly disagree, to 7, strongly agree) to assess perceived usefulness and ease of use. Study data were collected and managed using REDCap electronic data capture tools hosted at Geneva University Hospitals[8, 9]. REDCap (Research Electronic Data Capture) is a secure, web-based software platform designed to support validated data capture, audit trails, and seamless export to statistical software.

© 2026 Siebert JN et al. *JAMA Network Open.*

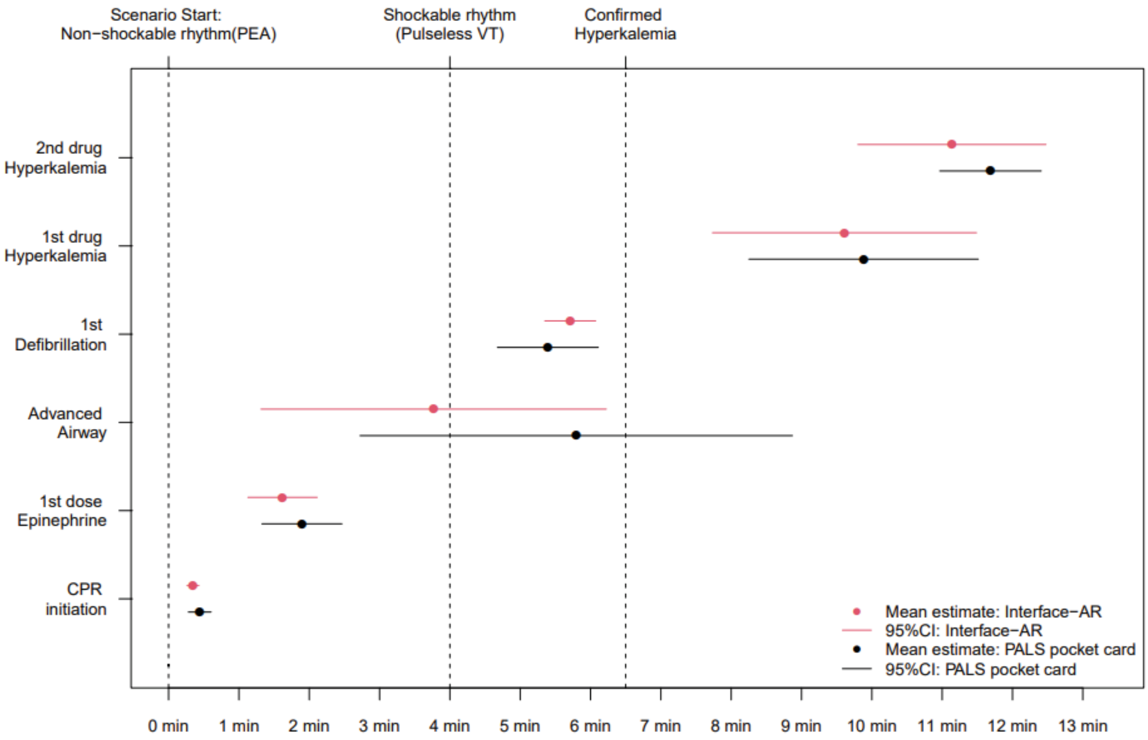

eFigure 1. Timing of critical resuscitation actions relative to AHA PALS targets

Mean time from scenario start to completion of each action with 95% confidence intervals for teams using Interface-AR (red) versus a PALS pocket card (black) during a simulated pediatric in-hospital cardiac arrest. Vertical dashed lines mark scenario phases; initial non-shockable rhythm (PEA), transition to a shockable rhythm (pulseless VT), and confirmation of hyperkalemia. Actions include CPR initiation, first epinephrine dose, advanced airway placement, first defibrillation, and first and second hyperkalemia drugs. Horizontal lines denote deviation around the mean estimates.

© 2026 Siebert JN et al. *JAMA Network Open.*

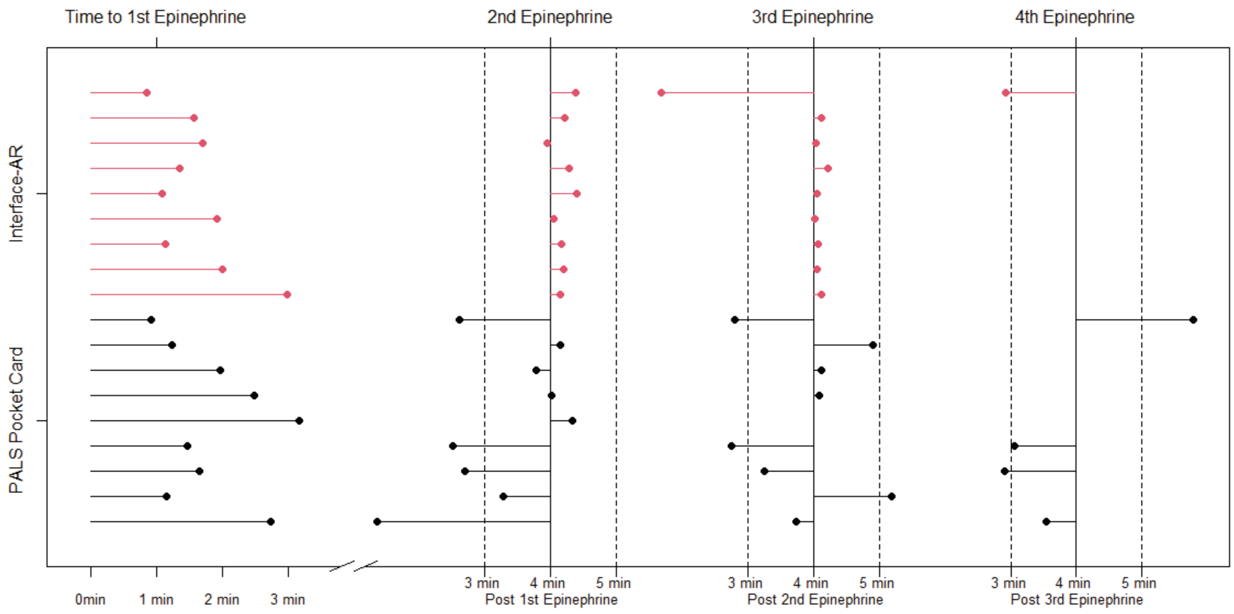

eFigure 2. Timing of sequential epinephrine doses and deviations from AHA recommended intervals

Each horizontal line represents one team's timing of epinephrine administrations for the InterFACE-AR (red) and PALS pocket card (black) groups. Columns correspond to the first through fourth epinephrine doses, with intervals measured from the previous dose. Dashed vertical lines indicate the AHA guideline–compliant interval of 3–5 minutes between subsequent doses.

© 2026 Siebert JN et al. *JAMA Network Open.*

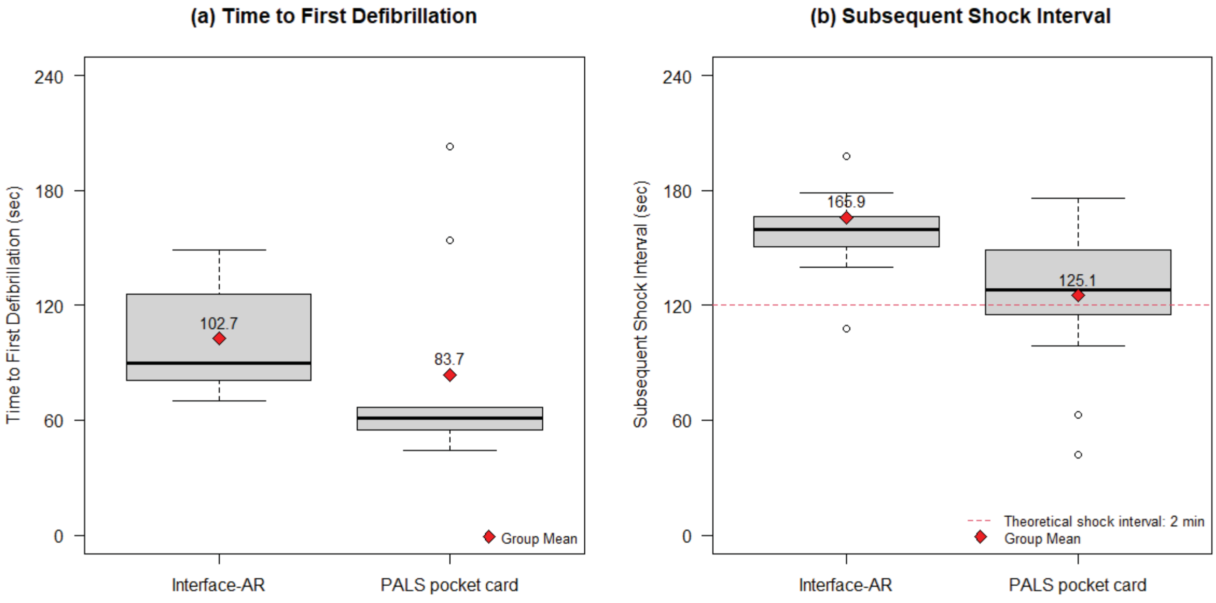

eFigure 3. Time to first and subsequent defibrillations, by study groups

Boxplots compare (left panel) time to first defibrillation and (right panel) intervals between subsequent shocks for teams using the InterFACE-AR system (intervention) versus the AHA PALS pocket card (control). The boxplots show the interquartile range (IQR), with whiskers extending to the most extreme values within 1.5×IQR from the quartiles. Horizontal lines indicate the median. The dots denote the mean values. The dashed red line denotes the theoretical 2-minute interval between defibrillations recommended by AHA guidelines.

© 2026 Siebert JN et al. *JAMA Network Open.*

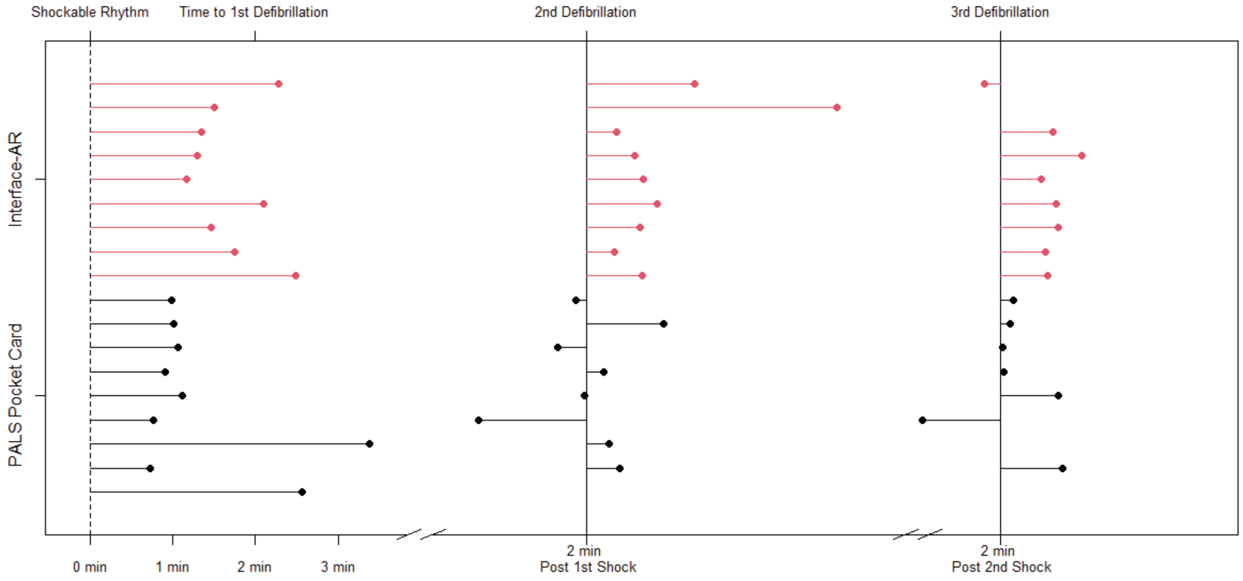

eFigure 4. Timing of sequential defibrillation

Each horizontal line represents one team's defibrillation timings for the InterFACE-AR (red) groups and PALS pocket card (black). The first column shows time to first defibrillation following onset of a shockable rhythm, while subsequent columns display intervals to the second and third defibrillations. Vertical dashed lines indicate the theoretical 2-minute interval between shocks recommended by AHA guidelines.

© 2026 Siebert JN et al. *JAMA Network Open.*

eTable 1. Medication dosing accuracy, by study group

Variables	Medication errors, n/N (%)		Risk ratio (95% CI)[a]	Risk difference (95% CI)[b]	*P* value[c]
	Intervention group (n = 9)	Control group (n = 9)			
Epinephrine dose deviation >10%	0/9 (0)	1/9 (11.1)	0.33 (0.02, 7.24)	-0.11 (-0.45, 0.22)	> 0.99
Amiodarone[d] dose deviation >10%	2/7 (28.6)	0/3 (0)	2.50 (0.15, 40.67)	0.29 (-0.40, 0.66)	> 0.99
Calcium dose deviation > 10%	2/6 (33.3)	0/7 (0)	2.86 (0.18, 45.91)	0.33 (–0.35, 0.72)	0.50

[a] Haldane–Anscombe correction applied for risk ratio calculation
[b] Newcombe's Method applied for 95% CI of risk difference
[c] Fisher's exact tests
[c] Used as the antiarrhythmic therapy for refractory VF/pVT

© 2026 Siebert JN et al. *JAMA Network Open.*

eTable 2. User experience questionnaire

Construct	Mean ± SD	Cronbach Alpha
Attractiveness	1.7 ± 1.0	0.937
Perspicuity	1.7 ± 1.0	0.839
Efficiency	1.8 ± 0.8	0.619
Dependability	1.7 ± 1.0	0.829
Stimulation	1.8 ± 1.1	0.881
Novelty	2.3 ± 0.7	0.589

© 2026 Siebert JN et al. *JAMA Network Open.*

eTable 3. Technology acceptance model

Construct	Mean ± SD	Cronbach Alpha
Perceived Usefulness	5.6 ± 1.4	0.976
Perceived Ease of Use	5.7 ± 1.4	0.958

© 2026 Siebert JN et al. *JAMA Network Open.*

eReferences

1. Ehrler F, Del Zotto M, Rouyer F, Weinhold T, Lovis C, Siebert J. Design of InterFACE: a tool to improve collaborative work and decision making during rescucitation. *Stud Health Technol Inform*. 2018;255:117-121.
2. Ehrler F, Sahyoun C, Manzano S, et al. Impact of a shared decision-making mHealth tool on caregivers' team situational awareness, communication effectiveness, and performance during pediatric cardiopulmonary resuscitation: study protocol of a cluster randomized controlled trial. *Trials*. Apr 13 2021;22(1):277. doi:10.1186/s13063-021-05170-3
3. Siebert JN, Lacroix L, Cantais A, Manzano S, Ehrler F. The impact of a tablet app on adherence to American Heart Association guidelines during simulated pediatric cardiopulmonary resuscitation: randomized controlled trial. *J Med Internet Res*. May 27 2020;22(5):e17792. doi:10.2196/17792
4. Rajic A, Olanka S, Generlli M, et al. Evaluating a shared decision support tool for pediatric cardiopulmonary arrest: a mixed methods usability study. *JMIR Hum Factors*. 2025;doi:10.2196/78736
5. Kang R, Cheng A, Lin Y, et al. Design, development, and usability evaluation of a role-specific augmented reality decision support system for cardiac arrest resuscitation. *JMIR Preprints*. 2025;doi:10.2196/preprints.72013
6. Schrepp M, Thomaschewski J, Hinderks A. Construction of a benchmark for the user experience questionnaire (UEQ). 2017;
7. King WR, He J. A meta-analysis of the technology acceptance model. *Information & management*. 2006;43(6):740-755.
8. Harris PA, Taylor R, Minor BL, et al. The REDCap consortium: building an international community of software platform partners. *J Biomed Inform*. Jul 2019;95:103208. doi:10.1016/j.jbi.2019.103208
9. Harris PA, Taylor R, Thielke R, Payne J, Gonzalez N, Conde JG. Research electronic data capture (REDCap) — a metadata-driven methodology and workflow process for providing translational research informatics support. *J Biomed Inform*. Apr 2009;42(2):377-81. doi:10.1016/j.jbi.2008.08.010

© 2026 Siebert JN et al. *JAMA Network Open.*
