# Supplementary material for: Augmented Reality–Guided Decision Support in Simulated Pediatric Cardiac Arrest: A Randomized Clinical Trial
Source: JAMA Netw Open. 2026 May 22;9(5):e2614030. doi: 10.1001/jamanetworkopen.2026.14030 (PMC13197869; doi:10.1001/jamanetworkopen.2026.14030)
Supplement: Supplement 3. — Data Sharing Statement [file jamanetwopen-e2614030-s003.pdf]

Data Sharing Statement

Siebert. Augmented Reality–Guided Decision Support in Simulated Pediatric Cardiac Arrest. *JAMA Netw Open*. Published May 22, 2026. doi:10.1001/jamanetworkopen.2026.14030

Data
Additional Information: ClinicalTrials.gov., https://clinicaltrials.gov/study/NCT06376643?cond=cardiac%20arrest&term=siebert&rank=1, NCT06376643
Data available: Yes
Data types: Deidentified participant data
How to access data: Deidentified participant data to replicate the primary analyses will be made available upon reasonable request to the author (Johan N. Siebert: johan.siebert@hug.ch).
When available: With publication

Supporting Documents
Document types: None

Additional Information
Who can access the data: Data will be made available to qualified external researchers whose proposed use of the data has been approved by their Institutional Review Board.
Types of analyses: Data will be accessible exclusively for research-related purposes.
Mechanisms of data availability: Data will be made available upon approval of a proposal and with a signed data access agreement.
Any additional restrictions: The request proposal must include a statistician.
